# Biocontrol mechanisms of two *Paenibacillus* strains against *Astragalus membranaceus* root rot and their effects on soil microecological structure

**DOI:** 10.3389/fmicb.2026.1827299

**Published:** 2026-06-01

**Authors:** Chenying Wu, Yue Lou, Litao Wang, Fan Wang, Xu Wang, Yang Liu, Constantine Uwaremwe, Zhaoyu Li, Zheng Zhang, Yonghong Zhu, Xu Su, Yongqiang Tian

**Affiliations:** 1 Key Laboratory of Microbial Resources Exploitation and Utilization of Gansu Province, Department of Microbiology, School of Biology and Pharmaceutical Engineering, Lanzhou Jiaotong University, Lanzhou, Gansu, China; 2 Gansu Pharmaceutical Group Technology Innovation Research Institute Co., Ltd., Lanzhou, Gansu, China; 3 Key Laboratory of Biodiversity Formation Mechanism and Comprehensive Utilization of the Qinghai-Tibet Plateau, Qinghai Normal University, Xining, Qinghai, China

**Keywords:** biocontrol, metagenome, *Paenibacillus polymyxa*, *Paenibacillus terrae*, promote plant growth, root rot disease

## Abstract

Astragalus membranaceus is an important medicinal herb in China, yet its yield and quality are severely constrained by root rot disease. In this study, two efficient antagonistic strains, HQ-1 and HQT-2, were isolated and identified as *Paenibacillus polymyxa* and *Paenibacillus terrae*, respectively. Both strains exhibited multiple plant growth-promoting traits and strong inhibitory activity against *Fusarium solani* (syn. *Neocosmospora solani*) GF-3. *In vitro* assays confirmed that their sterile fermentation filtrates effectively inhibited pathogen growth and damaged fungal hyphae. GFP labeling further verified their colonization potential on plant roots, while greenhouse experiments indicated preventive efficacies of 86.046% for HQ-1 and 80.619% for HQT-2. In addition, they significantly promoted the growth of *A. membranaceus*. Metagenomic analysis showed that biocontrol bacterium-treated soils had significantly increased relative abundance of beneficial microorganisms, alongside a reduction in phytopathogenic taxa. Notably, despite the scarcity of biocontrol reports for *P. terrae*, our study introduces strains HQ-1 and HQT-2 as highly effective, multifunctional resources for sustainable control of *A. membranaceus* root rot. This study provides much-needed systematic evidence on the efficacy of *P. terrae* in biocontrol, thereby addressing a notable lack of comprehensive data in the current literature.

## 1 Introduction

*Astragalus membranaceus* var. mongholicus (Bunge) P.K. Hsiao is a perennial leguminous herb widely distributed in China, mostly in the northwestern regions (Fan et al., 2025). As a principal medicinal herb, it contains a variety of active compounds such as polysaccharides, saponins, flavonoids and amino acids, which can promote antibody production, enhance immune responses, and support intestinal health (Su et al., 2023). Increasing market demand has driven the expansion in both artificial and wild-simulated cultivation of *A. membranaceus*. However, intensive production practices, including indiscriminate overuse of agrochemicals and irrational continuous cropping, have degraded soil structure, depleted organic matter, and fostered soil-borne diseases in traditional growing regions, seriously affecting both yield and quality (Li et al., 2022). Among these, root rot is a critical factor affecting the yield and quality of *A. membranaceus* (Wei et al., 2020). The incidence rate of root rot in *A. membranaceus* ranges from 35 to 92% in Dingxi City, Gansu Province (Zhang et al., 2025). After infection by pathogenic fungi, *A. membranaceus* not only suffers a substantial yield reduction but also experiences a decrease of approximately 30–40% in the contents of various endogenous nutrients (Zhang, 2024).

*Fusarium* is widely recognized as one of the most destructive soil-borne pathogens, causing root rot in numerous crops and resulting in serious production losses (Li X. P. et al., 2025; Jeffrey, 2016). According to literature reports, multiple species of the genus *Fusarium*, such as *Fusarium solani*, *Fusarium oxysporum*, and *Fusarium acuminatum*, can all infect *A. membranaceus* and cause root rot (Wang et al., 2025). Plants of *A. membranaceus* infected with root rot exhibit leaf chlorosis and wilting due to water loss. Symptoms include stems turning pale yellow, taproots developing reddish-brown dry rot, and root surfaces becoming rough, blackened and ulcerated, eventually leading to the death of the entire plant (Zhang X. C. et al., 2024). Furthermore, *Fusarium* species can infect a wide range of Chinese medicinal herbs. For instance, *F. oxysporum* can cause root rot in *Polygonatum kingianum* in Yunnan (Shang et al., 2025) and *Scutellaria baicalensis* in Gansu (Jiang et al., 2023), while *F. solani* can cause root rot in *Panax notoginseng* in Yunnan (Wang, 2014), resulting in significant economic losses. Although the pathogens causing *A. membranaceus* root rot have been known for some time, research on its biological control remains relatively scarce. Given the widespread impact and substantial economic losses associated with *Fusarium*-induced root rot, developing efficient and environmentally sustainable control strategies is imperative.

Biological control is one of the most promising alternatives to chemical control and is also recognized as an important measure for managing root rot (Lin, 2025). Biocontrol bacteria can directly inhibit pathogens through niche competition or production of antimicrobial substances. Furthermore, they can promote plant growth, elicit plant resistance, and even enhance soil quality (Jia et al., 2025). Numerous biocontrol bacteria have been recognized as important tools for controlling root rot. For instance, strains of the genera *Bacillus*, *Pseudomonas* and *Rhizobium* can inhibit the mycelial growth of *Fusarium solani*, thereby controlling soybean *Fusarium* wilt and root rot while simultaneously improving soybean yield through plant growth-promoting (PGP) properties (Kalantari et al., 2018). *B. velezensis* (strain Ba-0321) and *Paenibacillus polymyxa* (strain LB-9) produce broad-spectrum antimicrobial substances that antagonize *F. oxysporum* (Li et al., 2023). Remarkably, *P. polymyxa* exhibits notable biocontrol potential. It produces plant growth-promoting phytohormones indole-3-acetic acid (Khan et al., 2020), diterpenoids (Liu et al., 2020), and aids nutrient uptake through nitrogen fixation and phosphorus/potassium solubilization. Its safety profile has led to recognition as a commercializable microorganism by the US EPA (Wang and Liu, 2008). Huang et al. found that the strain *P. polymyxa* ZF197 effectively controlled Chinese cabbage stem base rot by secreting antimicrobial active substances (Huang et al., 2020). Similarly, Xu demonstrated that the strain *P. polymyxa* D-4 significantly inhibited the mycelial growth of *F. fujikuroi*, laying a foundation for the development of biocontrol bacteria targeting this pathogen (Xu, 2025). Likewise, *Paenibacillus terrae* exhibits good stability under light, pH and UV stresses, and produces antifungal compounds that inhibit fungal growth. Its fermentation metabolites damage fungal cell wall integrity and membrane permeability and reduce the content of pathogenicity-related factors (Yan, 2020). Therefore, this study adopts a biological control strategy, providing a promising new direction for the effective control of root rot in *A. membranaceus*.

Despite increasing interest in biological control of soil-borne diseases, effective microbial agents for managing of *A. membranaceus* root rot remain limited, and evidence integrating antagonistic activity, root colonization, plant growth promotion, and rhizosphere microecological responses is still insufficient. Although *Paenibacillus* species are widely recognized for their biocontrol and plant-beneficial potential, their application for controling *A. membranaceus* root rot and the associated effects on rhizosphere microbial structure have not been adequately characterized. Thus, the objectives of this study were: (1) to screen for highly efficient biocontrol bacteria against *F. solani*; (2) to evaluate the biocontrol characteristics of the efficient antagonistic strains and analyze their effectiveness in controlling root rot in *A. membranaceus*; and (3) to explore whether inoculation with these strains is associated with changes in the composition and functional potential of the rhizosphere microbiome. This work is expected to provide candidate microbial resources for the development of multifunctional biocontrol agents and to offer a basis for understanding the ecological processes associated with disease suppression in *A. membranaceus*.

## 2 Materials and methods

### 2.1 Isolation and identification of the pathogen causing root rot

#### 2.1.1 Field sampling, isolation and purification of pathogens

In October 2023, samples of *A. membranaceus* with root rot were collected in Min County, Gansu Province. Three sampling areas were set in the field, from which healthy plants and plants with typical root rot symptoms were collected, respectively. Diseased plants mainly showed yellowing leaves, rotting roots and plant death. At each sampling site, 3 diseased plants and 1 healthy plant were randomly selected, resulting in a total of 12 root samples. Rhizosphere soil samples of healthy plants were also collected at the same time. All samples were stored at low temperatures and transported back to the laboratory for pathogens isolation and purification.

After washing the diseased roots, severely rotted tissues were disinfected with 70% ethanol and 3% sodium hypochlorite, rinsed with sterile water, and then inoculated onto PDA medium. A total of 0.5 g of rhizosphere soil was shaken with sterile water and diluted in a series of 10^–1^–10^–5^ dilutions. Aliquots of 100 μL from each dilution were spread on PDA plates with six replicates. All plates were incubated at 25°C for 5–7 days. If contaminated, mycelia at the colony edge were picked and purified until pure strains were obtained.

#### 2.1.2 Pathogenicity assay

An *in vitro* inoculation method was used to verify the pathogenicity of the isolated strains. Healthy Astragalus roots with uniform growth were surface-disinfected, pricked, and placed in sterile Petri dishes with moist absorbent cotton for humidity maintenance. Mycelial plugs of 5 mm were inoculated onto root wounds, while sterile agar plugs were used as the control. All treatments were incubated at 25°C until typical lesions appeared, with three biological replicates. After incubation, the pathogen was re-isolated from diseased tissues to fulfill Koch’s postulates.

#### 2.1.3 Morphological and molecular identification

Pathogens were identified by morphological and molecular methods. Preliminary classification was performed by observing colony morphology, pigment production, and microscopic characteristics of mycelia and spores on PDA medium. Total DNA was extracted from fresh mycelia using a fungal DNA kit. The ITS region and EF1-α gene were amplified with primer pairs ITS1/ITS4 and EF1/EF2, respectively. PCR products were sequenced, and the sequences were compared in the NCBI database. A phylogenetic tree was constructed using the neighbor-joining method in MEGA 11.0 to determine the taxonomic status of the pathogen.

### 2.2 Experimental strains, culture medium, and pot experiment conditions

Required strains preserved in the laboratory were cultured on potato dextrose agar (PDA) at 30°C for 20 h prior to use. Those strains include (1) *Fusarium solani* GF-3, (2) *Botrytis cinerea*, (3) *Fusarium graminearum*, (4) *Fusarium acuminatum*, (5) *Fusarium equiseti*, (6) *Fusarium avenaceum*, (7) *Rhizoctonia solani*.

The plasmid pGFP4412 utilized in this study was obtained from Fenghui Biotech. The media and their respective compositions utilized in this study are presented in the Supplemental Materials. All pot experiments were conducted in a greenhouse facility at Lanzhou Jiaotong University from May to June 2025, using only *Astragalus membranaceus* var. *mongholicus* (Bunge) P.K.Hsiao.

### 2.3 Screening and identification of biocontrol bacteria

Healthy *A. membranaceus* roots were surface-sterilized, cut into small pieces, and ground into homogenate. The homogenate was collected and diluted in sterile water to 3 concentration gradients: 10^–5^, 10^–6^, and 10^–7^, with 6 replicates per dilution. A 100 μL aliquot of each sample was evenly spread onto LA and PDA media, and the plates were incubated at 25 °C for 3–4 days. Distinct colonies were picked and purified until axenic cultures were obtained.

The *in vitro* antagonistic activity of the isolated biocontrol strains against the pathogenic fungi was evaluated using the dual-culture plate assay. The target pathogenic fungus isolate and each test bacterium were simultaneously inoculated onto PDA medium using a three-point confrontation method. Plates inoculated with the pathogen alone served as the blank control, with three replicates per treatment. All plates were incubated at 28 °C in a fungal incubator until the mycelia in the control group fully covered the plate surface. The inhibition rate of each strain was calculated using the following formula:


$$\mathrm{Inhibition}\mathrm{rate}\left(\%\right)=\frac{\mathrm{colony}\,\mathrm{diameter}\,\mathrm{of}\,\mathrm{control}-\mathrm{colony}\,\mathrm{diameter}\,\mathrm{of}\,\mathrm{treatment}}{\mathrm{colony}\,\mathrm{diameter}\,\mathrm{of}\,\mathrm{control}}\times 100\%$$

The screened biocontrol strains were subjected to Gram staining, and the genomic DNA was extracted from the selected strains using a Bacterial DNA Rapid Extraction Kit. Molecular identification was performed by amplifying the nearly full-length 16S rRNA gene using the universal bacterial primers 27F (5’-AGAGTTTGATCCTGGCTCAG-3’) and 1492R (5’-GGTTACCTTGTTACGACTT-3’). Additionally, the DNA gyrase subunit B gene was amplified using the specific primers gyrB-F (5’-GAAGTCATCATGACCGTTCTGCA-3’) and gyrB-R (5’-AGCAGGGTACGGATGTGCGAGCC-3’) for further phylogenetic analysis. PCR amplification was performed under the following conditions: initial denaturation at 94°C for 5 min; 30 cycles of denaturation at 94°C for 30 s, annealing at 51°C for 45 s, and extension at 72°C for 1 min; and a final extension at 72°C for 10 min. Amplicons were purified and sequenced by Beijing Tsingke Biotech Co., Ltd. A neighbor-joining (NJ) phylogenetic tree was constructed with MEGA 11.0.

### 2.4 Determination of plant growth promotion characteristics

The IAA-producing capacity was determined using the modified Salkowski colorimetric method (Guardado-Fierros et al., 2024). The cultures of HQ-1 and HQT-2 were placed in King’s medium with and without L-tryptophan (Macklin, Shanghai, China) for 2 days at 30°C and 180 rpm. After centrifugation at 5,000 rpm for 5 min, the supernatant was mixed with Salkowski’s reagent in a 1:1 ratio using brief vortexing. After incubation at 30°C in the dark for 30 min, the absorbance of the mixture was measured at 530 nm using a spectrophotometer. The IAA concentration was quantified by comparing these readings to a standard IAA solution (Sigma-Aldrich, Burlington, MA, United States).

In addition, the strains were inoculated onto specific selective media for detecting phosphate dissolution, potassium solubilization, siderophore production and nitrogen fixation, followed by incubation at 28°C for 5 days. A positive result was recorded if a transparent halo formed around the colonies (Wang Y. H. et al., 2022). Nitrogen fixation capacity was verified by the growth of colonies on nitrogen-free medium (Li et al., 2021). The ratio of the transparent halo diameter to the colony diameter was defined as the D value, and the presence or absence of transparent halos and the magnitude of the D value can be used as criteria for evaluating PGP activity (Wu et al., 2026; Yang et al., 2023).


$$D\,\mathrm{va}\,\text{lue}=\frac{\mathrm{transparent}\,\mathrm{halo}\,\mathrm{diameter}}{\mathrm{colony}\,\mathrm{diameter}}$$

### 2.5 Determination of biofilm formation

Biocontrol bacteria were cultured to an OD_600_ = 1 at 28°C and 180 rpm. Each well of a 24-well plate received 1.8 mL sterile Potato Dextrose Broth (PDB) plus 0.2 mL bacterial culture; controls received 2 mL sterile PDB. After 48 h at 37°C, the medium was removed, wells were washed three times with PBS, dried at room temperature or under airflow, and 200 μL 0.1% crystal violet was added for 30 min. Wells were rinsed with PBS until transparent, then 95% ethanol was added and left for 30 min; absorbance at OD_590_ was measured.

The cut-off value (ODc) was defined as the mean OD of the control plus three times its standard deviation (Cao et al., 2025). Strains were classified as: OD590 ≤ ODc, non-biofilm-forming strain (-); ODc < OD_590_ ≤ 2 ODc, weak biofilm-forming strain (+); 2 ODc < OD_590_ ≤ 4 ODc, moderate biofilm-forming strain (++); OD_590_ > 4 ODc, strong biofilm-forming strain (+++).

### 2.6 Colonization effect of biocontrol bacteria

To observe the root colonization of *A. membranaceus* by strains HQ-1 and HQT-2, green fluorescent protein (GFP)-labeled derivatives GFP-HQ-1 and GFP-HQT-2 were constructed using the GFP vector pGFP4412 (Xiong et al., 2020). Prior to GFP-based colonization experiments, preliminary laboratory tests were conducted to evaluate the growth characteristics and marker stability of the GFP-tagged strains. These tests indicated that GFP-HQ-1 and GFP-HQT-2 exhibited growth rates comparable to those of the wild-type strain and maintained stable fluorescence during serial subculturing under laboratory conditions (Ma, 2022). For colonization assays, the GFP-tagged strain was inoculated in 100 mL of PDB supplemented with 100 μg⋅mL^–1^ ampicillin and incubated overnight at 30°C. Vigorously growing *A. membranaceus* were surface sterilized at the roots and then transplanted into pots containing sterilized soil. Each seedling was root-drenched with 100 mL of the GFP MM suspension (10^9^ CFU⋅mL^–1^), while the control group received 100 mL of sterile water. All treated plants were maintained at a constant temperature of 25°C (Cao et al., 2011). At 4, 8, 12, 16, and 20 dpi, stem and root samples were randomly collected. At each sampling time point, different plants were selected and destructively sampled. Colonization was observed under a laser confocal scanning microscope (excitation wavelength = 488 nm). For quantification, 0.1 g of root samples was ground in 0.9 mL of sterile water, then diluted in a series and plated onto 100 μg/mL ampicillin-containing selective agar for their respective CFU enumeration (Cui et al., 2019). Prior to statistical analysis, CFU data were log10-transformed [log10 (CFU + 1)], as bacterial population data typically do not follow a normal distribution.

### 2.7 Inhibitory effect of sterile filtrate of biocontrol bacteria on the pathogen

Single colonies of HQ-1 and HQT-2 were picked and inoculated separately into PDB medium, then incubated with shaking at 30°C and 180 rpm for 12 h to prepare overnight cultures. Cells were then harvested by centrifugation at 5,000 rpm for 10 min. The resulting supernatant was aseptically filtered through a 0.22 μm membrane to obtain a cell-free filtrate. PDA plates supplemented with 10, 30, and 50% (v/v) fermentation filtrate were prepared by mixing the sterile filtrate with molten PDA medium at volume ratios of 1:9, 3:7, and 1:1, respectively. A mycelial plug of GF-3 was then inoculated at the center of each plate. All plates were then incubated at 25°C. The PDA plates without the fermentation filtrate served as the control. When the mycelial growth in the control plates reached the plate margins, data were collected. The inhibition rate for each biocontrol bacterium was calculated using the following formula:


$$\mathrm{Inhibition}\mathrm{rate}\left(\%\right)=\frac{\mathrm{colony}\,\mathrm{diameter}\,\mathrm{of}\,\mathrm{control}-\mathrm{colony}\,\mathrm{diameter}\,\mathrm{of}\,\mathrm{treatment}}{\mathrm{colony}\,\mathrm{diameter}\,\mathrm{of}\,\mathrm{control}}\times 100\%$$

### 2.8 Disruption of pathogen hyphae by biocontrol bacteria

GF-3 mycelial plugs (*n* = 6) were incubated in PDB for 72 h. Harvested mycelia were treated with sterile cell-free filtrates of the two biocontrol strains for 10 h, washed thrice with PBS (pH 7.2), and resuspended. A 2-mL aliquot of the suspension was fixed in 2.5% glutaraldehyde for 4 h. After centrifugation and removal of the supernatant, the pellets were washed four times with PBS, recentrifuged, and subjected to serial dehydration in a graded ethanol series (30, 50, 70, 90, and 100%) for 15 min each, with a final 15-min incubation in 100% ethanol. Samples were stored at -80°C for 8–10 h, then freeze–dried under vacuum for 18 h. Finally, morphological observations were performed using a scanning electron microscope (Zeiss Gemini SEM 500, Germany), with three experimental replicates (Zhao and Hao, 2020).

To assess the disruptive impact of the HQ-1 and HQT-2 on GF-3 cell membranes, changes in membrane permeability were evaluated by measuring extracellular conductivity (Wang et al., 2019). Five pathogen plugs were inoculated into 100 mL PDB and incubated in a constant-temperature shaker for 4 d to obtain mycelia. The mycelia were washed three times with 10 mL sterile PBS and resuspended in 100 mL fresh PBS. Sterile filtrate was added at 10, 20, or 50% (v/v); controls received an equal volume of PBS containing the same amount of PDB. The suspensions were shaken at 28°C, and membrane conductivity was measured every 3 h with a conductivity meter; each treatment was repeated three times.

### 2.9 *In vivo* assay for biocontrol of *A. membranaceus* root rot

To evaluate the *in vivo* potential of the two biocontrol bacteria to suppress *A. membranaceus* root rot, a greenhouse trial was conducted. One-year-old healthy *A. membranaceus* seedlings were grown in pots containing a 1:1 (v/v) mixture of autoclaved black soil and nutrient-rich soil for 7 days before inoculation. The GF-3 spore suspension was adjusted to a concentration of 105 CFU⋅mL^–1^ and used as the pathogen inoculum. The bacterial fermentation broths were adjusted to 10^8^ CFU⋅mL^–1^ and served as the two biocontrol inocula. This experiment was divided into eight treatment groups (two of which were dedicated to the pot experiment for plant growth promotion.), with three replicates per group and 10 plants per replicate. Before inoculation, a small wound approximately 5 mm in length was made on the surface of each taproot using a sterile scalpel. Treatments for the eight groups were performed as follows:

(1) Healthy Control (CK): each plant was irrigated twice with 30 mL of sterile PDB at an interval of 48 h.

(2) Pathogen Control (GF-3): each plant was irrigated twice with 30 mL of spore suspension at an interval of 48 h.

(3) HQ-1 only: each plant was irrigated twice with 30 mL of HQ-1 fermentation broth at an interval of 48 h.

(4) HQT-2 only: each plant was irrigated twice with 30 mL of HQT-2 fermentation broth at an interval of 48 h.

(5) Curative treatment 1: GF-3 + HQ-1: each plant was irrigated twice with 30 mL of spore suspension first; 48 h later, each plant was irrigated with 30 mL of HQ-1 fermentation broth.

(6) Curative treatment 2: GF-3 + HQT-2: each plant was irrigated twice with 30 mL of spore suspension first; 48 h later, each plant was irrigated with 30 mL of HQT-2 fermentation broth.

(7) Preventive treatment 1: HQ-1 + GF-3: each plant was irrigated twice with 30 mL of HQ-1 fermentation broths, respectively, at an interval of 48 h; 48 h later, each plant was irrigated with 30 mL of spore suspension.

(8) Preventive treatment 2: HQT-2 + GF-3: each plant was irrigated twice with 30 mL of HQT-2 fermentation broths, respectively, at an interval of 48 h; 48 h later, each plant was irrigated with 30 mL of spore suspension.

Subsequently, the plants were watered regularly once every 2 days. Disease severity (DS) was assessed 30 days post-inoculation. The DS was evaluated using the 0–7 grading scale for root rot of rhizome medicinal materials (Yang et al., 2025).

0: No disease; 1: Lesion area accounts for 1–5% of root surface area; 2: 6–10%; 3: 11–20%; 4: 21–40%; 5: 41–60%; 6: 61–80%; 7: > 80% of root surface area.

### 2.10 Effects of biocontrol bacteria on plant defense-related enzyme activities

After 30 days of the pot experiment, root tissues of *A. membranaceus* were collected separately from the CK group and the growth-promotion group, for the determination of the activities of four defense-related enzymes. For the subsequent determination of four defense-related enzyme activities, the samples were divided into 4 groups with 3 replicates per group. The assayed defense-related enzymes included phenylalanine ammonia-lyase (PAL), peroxidase (POD), catalase (CAT), and polyphenol oxidase (PPO). For each enzyme, its activity was quantified according to the protocol provided with its commercial assay kit (AIDISHENG, Jiangsu, China). The catalog numbers are ADS043TE0, ADS050TE0, ADS-W-KY002, and ADS052TE0 in sequence. Absorbance were measured with a spectrophotometer and a microplate reader to determine enzyme activity levels (Wang et al., 2020).

### 2.11 Determination of endogenous nutrients in *A. membranaceus*

After 30 days of treatment in the pot experiment, root tissues of *A. membranaceus* were collected from the CK and the growth-promotion groups. The samples were divided into 4 groups with 3 replicates per group, and sent to Nanjing Zhenke Testing Technology Co., Ltd. Total phenols were determined by the Folin-Ciocalteu colorimetric method, polysaccharides by the phenol-sulfuric acid method, flavonoids by the aluminum nitrate colorimetric method, and saponins by the methanol-sulfuric acid reflux extraction method.

### 2.12 The impact of biocontrol bacteria on soil microbial communities

To assess the impact of the biocontrol bacteria on the rhizosphere microbial communities, metagenomic sequencing was performed. At 30 days after treatment in the pot experiment, rhizosphere soils from the CK group and the two growth-promotion groups were collected. For each group, soil samples were randomly taken from 3 separate spots, mixed thoroughly, respectively, passed through a 2 mm sieve, and then each was divided into 3 equal aliquots, which served as 3 biological replicates. The aliquots were placed in sterile bags and sent to Biomarker Technologies (Beijing) for Illumina metagenomic sequencing. Soil sample DNA was extracted using the TIANMicrobe Magnetic Envir-DNA Kit 9. Libraries were constructed with the VAHTSTM Universal Plus DNA Library Prep Kit for Illumina. The PCR primer sequences were as follows: adapter3 = AGATCGGAAGAGCACACGTCTGAACTCCAGTCAC and adapter5 = AGATCGGAAGAGCGTCGTGTAGGGAAAGAGT GT. After sequencing was completed, bioinformatic analyses were performed to obtain information related to gene functions, species composition, and other environment-associated data (Wang et al., 2026).

All data analyses were performed on the Biomarker BioCloud Platform. Relevant analysis tools were used to calculate Alpha diversity indices, and *t*-tests were conducted to analyze inter-group differences in Alpha diversity. Beta diversity based on the Bray-Curtis distance was used to assess microbial community similarity among samples, and PERMANOVA nonparametric tests were used to determine the significance of differences in microbial community structure between sample groups. LEfSe analysis (LDA > 2, *p* < 0.05) was used to identify microbial taxa with significantly different abundances at the phylum-to-genus level across groups. For sequencing data processing, MEGAHIT was used for metagenomic assembly, and QUAST was used to evaluate the assembly results. Non-redundant gene sets were constructed using MMseqs2. Functional annotation covered general databases such as Nr, GO, and KEGG, as well as specialized databases including CAZy and CARD. Annotation information was obtained through BLAST alignment (*E*-value 1e-5) or via specialized software (e.g., HMMER, RGI).

### 2.13 Statistical analysis

All data in the experiment are presented as the mean ± standard deviation (SD) of three replicates, while the data from the pot biocontrol experiment are recorded from ten replicates. Nonparametric tests were used to calculate the median, mean rank, and relative treatment effect for each treatment group; the severity of GF-3 induced-root rot was assessed using the 95% confidence interval of the relative treatment effect (Mu, 2024). All figures in this paper were plotted using Origin 2024 software. Experimental data were statistically analyzed using SPSS 26.0; one-way analysis of variance (ANOVA) was used to compare differences among treatments, and significance was determined at α = 0.05.

## 3 Results

### 3.1 Isolation and pathogenicity assay of pathogens

**FIGURE 1:**
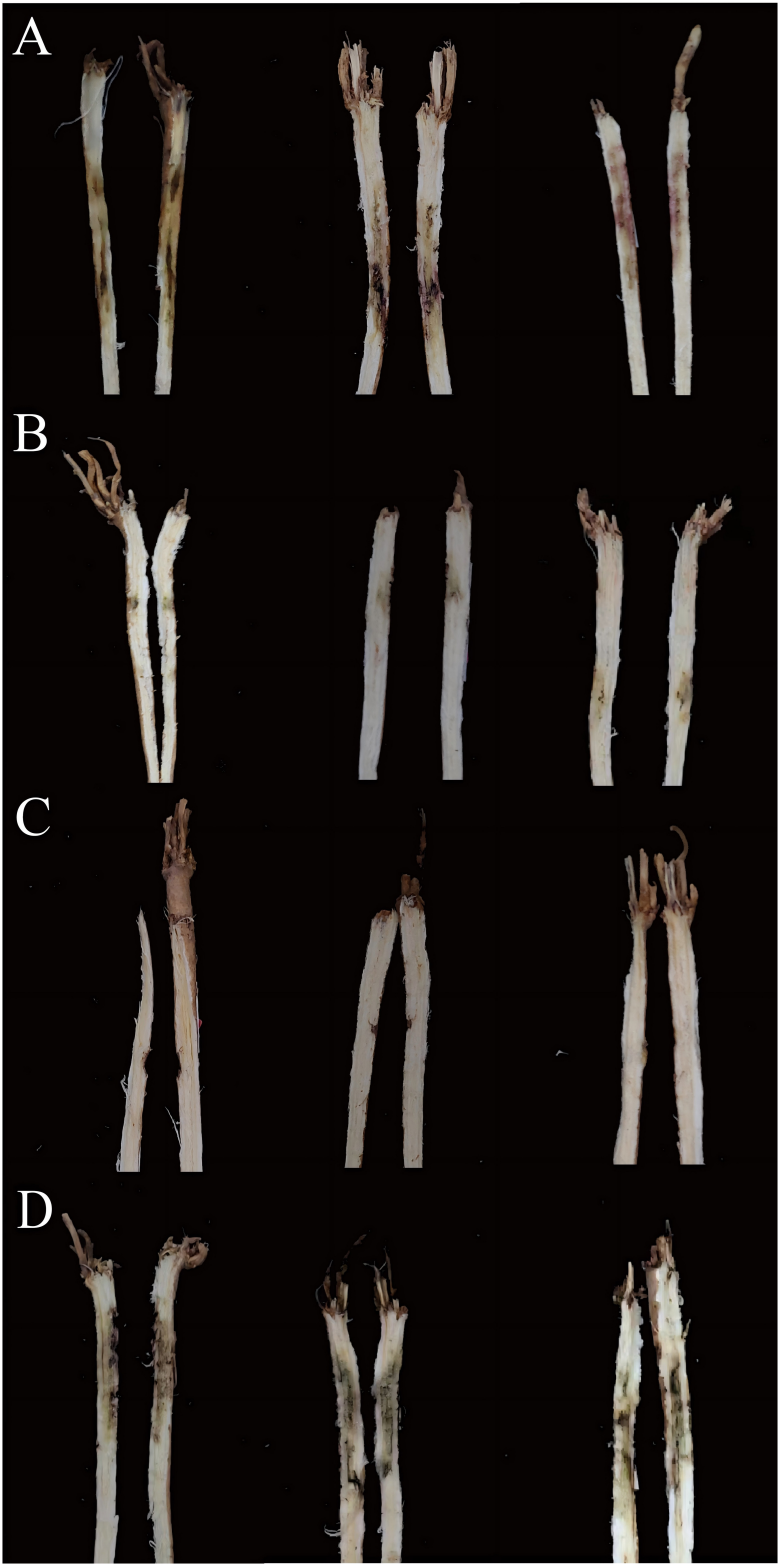
Pathogenicity assay. **(A)** CK; **(B)** GF-1 treatment group; **(C)** GF-2 treatment group; **(D)** GF-3 treatment group.

**FIGURE 2:**
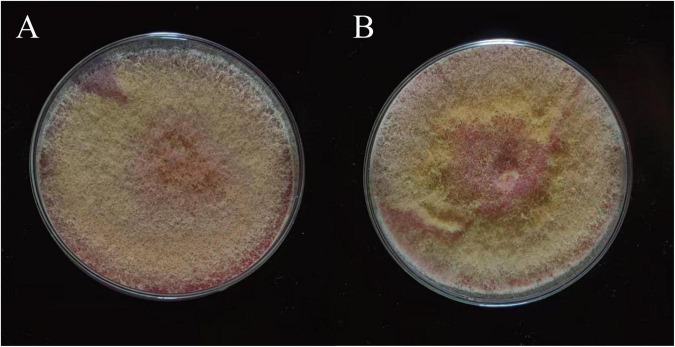
Comparison of isolates. **(A)** Original strain GF-3. **(B)** Isolated strain from the diseased group.

Three distinct strains, designated GF-1, GF-2, and GF-3, were isolated from diseased *A. membranaceus* roots based on morphological observations. The results of the *in vitro* pathogenicity assay are shown in Figure 1. Ten days after inoculation, the *A. membranaceus* tissues exhibited varying degrees of symptoms. Among them, tissues inoculated with strain GF-3 showed the most severe infection: the root epidermis was damaged, the internal tissues turned dark brown and rotted, consistent with symptoms of naturally diseased *A. membranaceus*. Therefore, strain GF-3 was identified as the causal agent of root rot in *A. membranaceus*. The strain was re-isolated from the roots of *A. membranaceus* in the GF-3 treatment group and was consistent with the original GF-3 strain, fulfilling Koch’s postulates (Figure 2).

### 3.2 Identification of the pathogen

**FIGURE 3:**
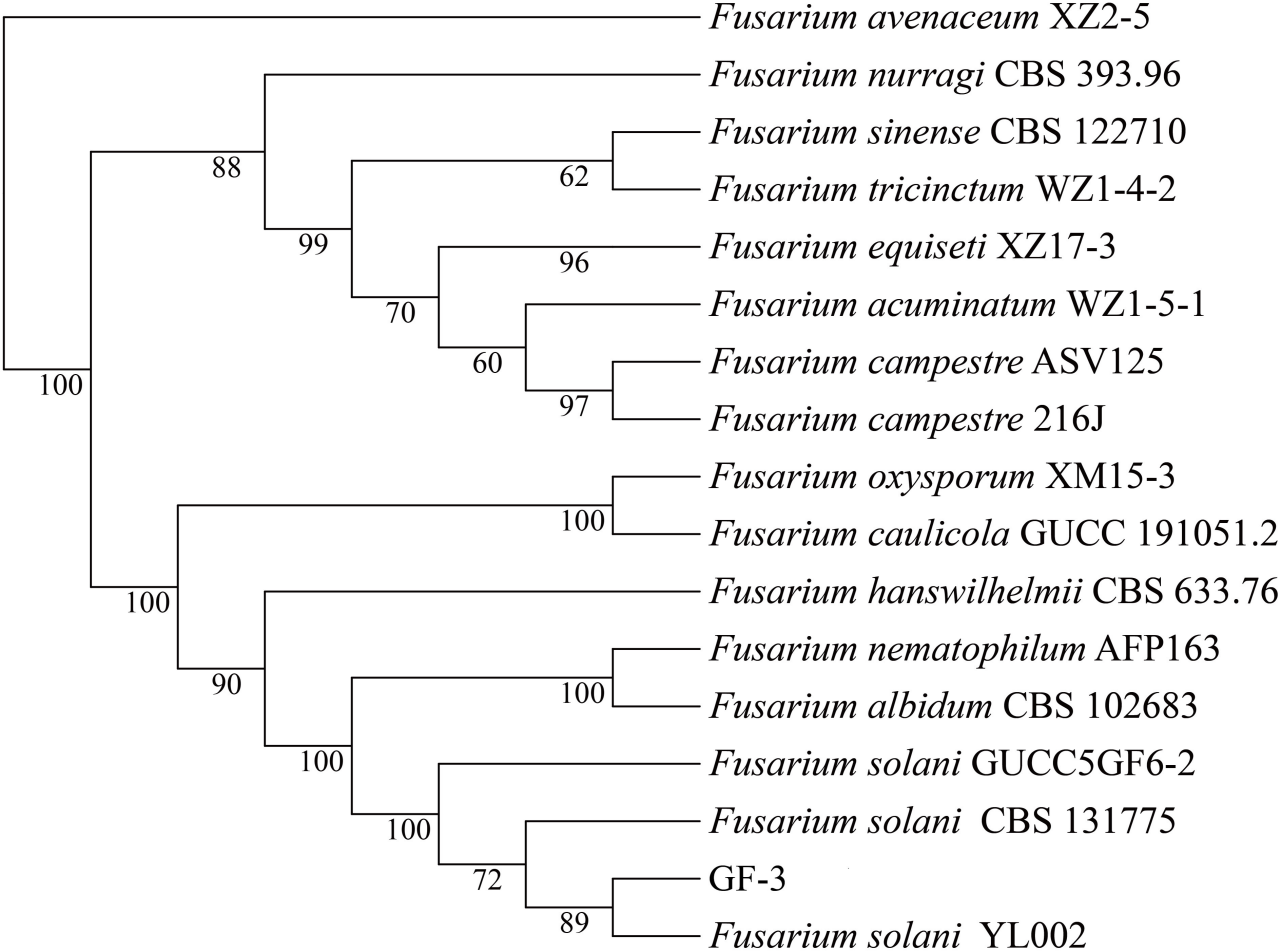
Morphological features of pathogen GF-3.

The ITS and EF1-α gene regions of the pathogen were amplified by PCR and sequenced. BLAST sequence alignment in NCBI confirmed that the isolate belongs to the genus *Fusarium* sp. A phylogenetic tree was constructed using closely related type strains. As shown in Figure 3, strain GF-3 clustered in the same phylogenetic clade as *Fusarium solani* (syn. *Neocosmospora solani*), by which the strain was clearly identified as *Fusarium solani* (syn. *Neocosmospora solani*). The gene sequences were deposited in GenBank under accession numbers PX844730.

### 3.3 Isolation, screening, and identification of biocontrol bacteria

**FIGURE 4:**
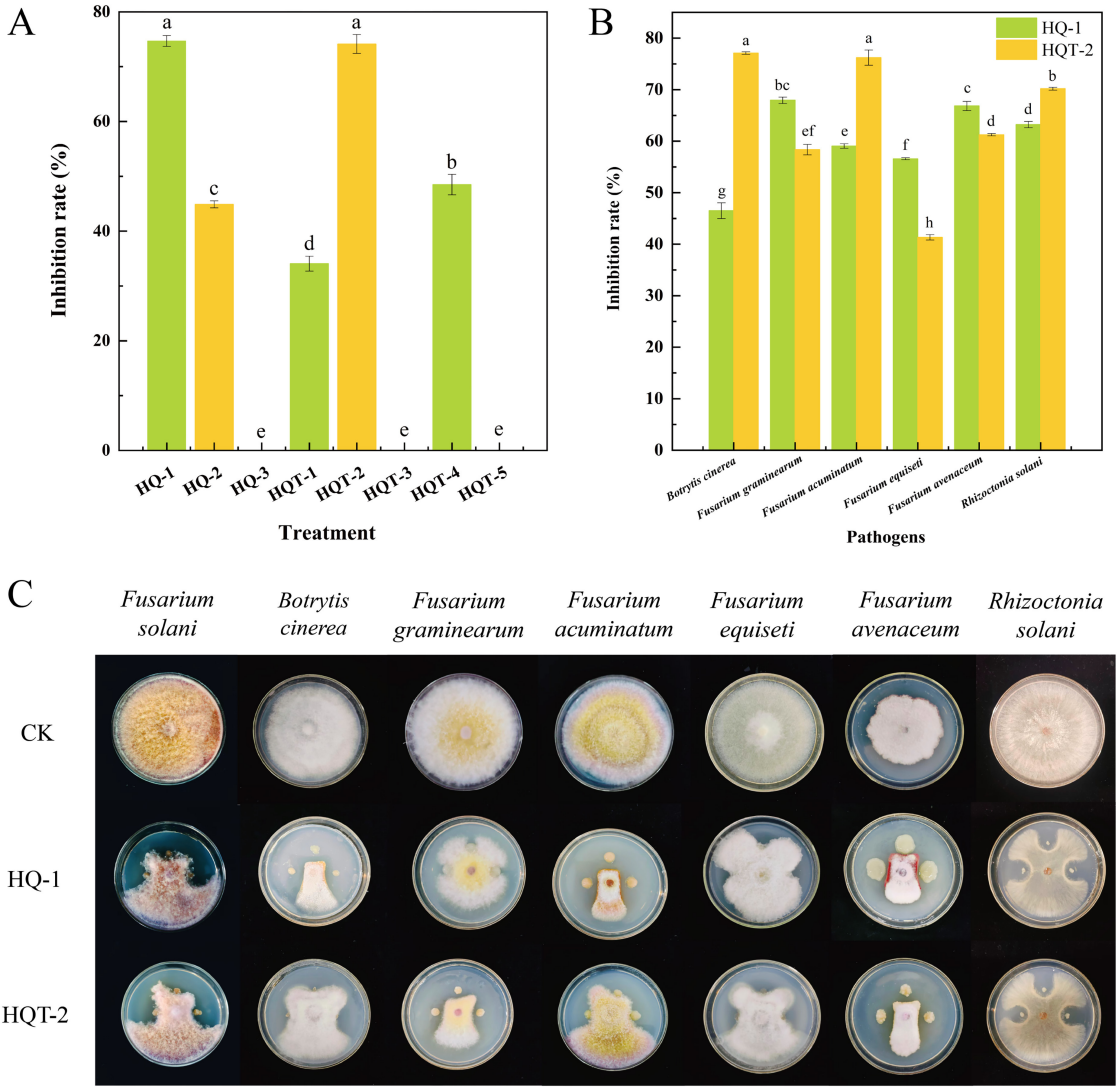
**(A)** Plate inhibition rates of 8 bacterial strains against GF-3. **(B)** Broad-spectrum inhibition rates of HQ-1 and HQT-2. **(C)** The first column shows the plate confrontation assay of HQ-1 and HQT-2 against GF-3, and the remaining columns display the broad-spectrum antagonistic plate confrontation assays of HQ-1 and HQT-2. Letters a–f above the bars indicate significant differences (*p* < 0.05) according to Tukey’s HSD test. Values with different letters are significantly different, while values with the same letter are not significantly different.

Strains were isolated from all three serial dilutions, with a total of 8 isolates, including 3 endophytic strains (HQ-1, HQ-2, HQ-3) and 5 rhizosphere strains (HQT-1, HQT-2, HQT-3, HQT-4, HQT-5), and their antagonistic activities against *F. solani* GF-3 are shown in Figure 4A (Tukey’s HSD, *p* < 0.05). Among them, strain HQT-2 isolated from soil and strain HQ-1 isolated from root displayed the most potent antagonistic activity against *F. solani* GF-3 (Figure 4C), with inhibition rates of 74.71% and 74.15 %, respectively (mean ± SD, *n* = 3). Broad-spectrum inhibition assays of strains HQ-1 and HQT-2 showed that both exhibited antagonistic activity against several common soil-borne pathogens (Tukey’s HSD, *p* < 0.05; Figures 4B,C). Based on morphological and molecular identification, HQ-1 and HQT-2 were identified as *Paenibacillus polymyxa* and *Paenibacillus terrae*, respectively (Supplementary Figures 1A,B), and both were Gram-positive (Supplementary Figures 1C,D). The 16S rRNA gene sequences were deposited in GenBank under accession numbers PX844714 and PX844720.

### 3.4 PGP characteristic assays

Both biocontrol strains were able to produced IAA. Upon supplementation with L-tryptophan, IAA production by both HQ-1 and HQT-2 was significantly increased, reaching 51.65 mg⋅L^–1^ and 47.09 mg⋅L^–1^, respectively (Supplementary Table 1). This confirms their strong potential for IAA biosynthesis.

**FIGURE 5:**
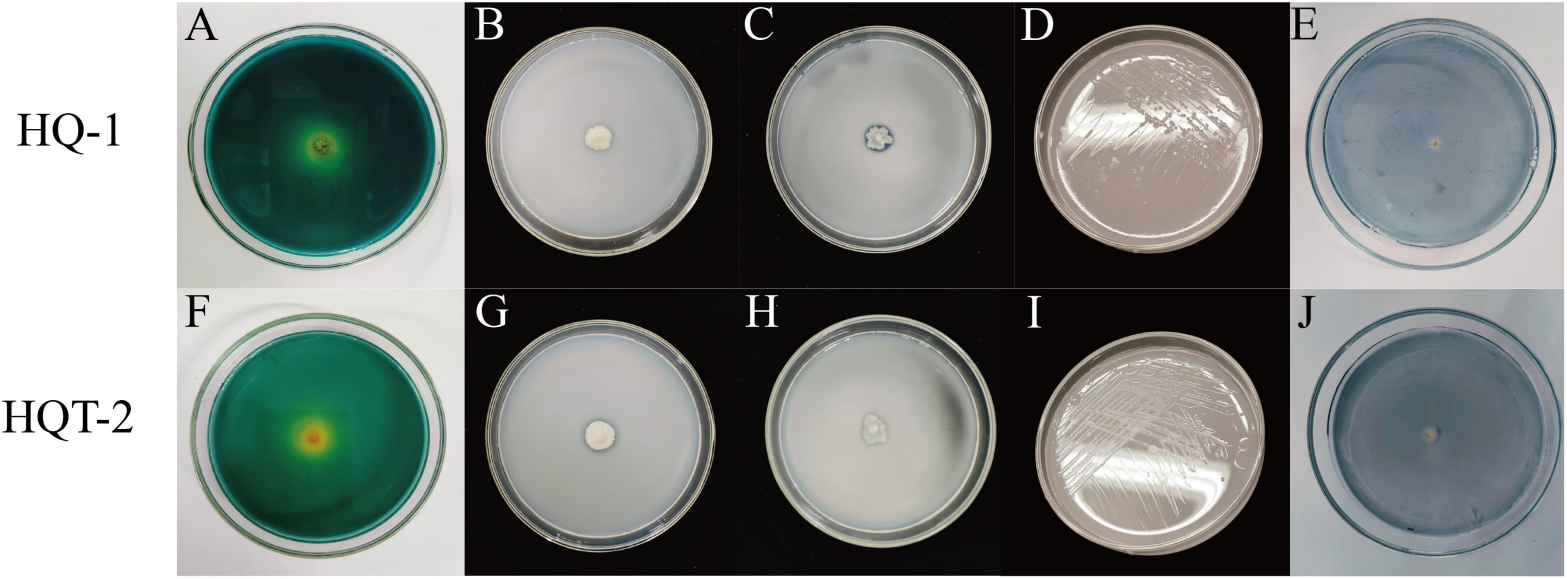
Plant growth-promoting characteristics of the two biocontrol bacteria. **(A,F)** Potassium-dissolving medium. **(B,G)** Phosphate-solubilizing medium; **(C,H)** Phosphate—dissolving medium; **(D,I)** Ashby’s nitrogen-free medium; **(E,J)** CAS medium.

Both strains formed clear halos on phosphate-solubilizing detection medium, potassium-dissolving detection medium, phosphate-dissolving detection medium and CAS detection medium, and grew well on Ashby’s Nitrogen-free medium, confirming their abilities to dissolve potassium, solubilize phosphate, dissolve phosphate, fix nitrogen and produce siderophore (Figure 5). Among them, HQT-2 had a D-value greater than 1.05 on the phosphate solubilization medium, indicating that it is a highly efficient phosphate-solubilizing bacterium (Wu et al., 2026; Tukey’s HSD, *p* < 0.05; Table 1).

**TABLE 1:** PGP-characteristic D values and activity levels of HQ-1 and HQT-2.

Plant growth-promoting characteristic	Treatment	Transparent circle diameter (cm)	Colony diameter (cm)	*D*-value
Potassium dissolution	HQ-1	1.807 ± 0.091	0.783 ± 0.042	2.308^ab^
	HQT-2	1.910 ± 0.036	0.573 ± 0.040	3.333^a^
Phosphate solubilization	HQ-1	1.317 ± 0.025	1.263 ± 0.065	1.043^b^
	HQT-2	1.453 ± 0.150	1.300 ± 0.184	1.118^ab^
Phosphate dissolution	HQ-1	1.250 ± 0.010	0.883 ± 0.029	1.416^ab^
	HQT-2	0.737 ± 0.095	0.626 ± 0.015	1.177^ab^
Siderophore production	HQ-1	0.433 ± 0.060	0.240 ± 0.036	1.804^ab^
	HQT-2	0.520 ± 0.122	0.333 ± 0.040	1.562^ab^
Nitrogen fixation	HQ-1	–	–	–
	HQT-2	–	–	–

Values are presented as mean ± SD (n = 3). Different lowercase letters within the same column indicate significant differences among treatments according to Tukey’s HSD test (*p* < 0.05).

### 3.5 Determination of biofilm-forming capacity and colonization ability

According to Supplementary Figure 2A, both biocontrol strains exhibited favorable biofilm-forming ability. Among them, *P. polymyxa* HQ-1 showed strong biofilm formation ( > 4OD_*C*_), while *P. terrae* HQT-2 was a moderate biofilm-forming strain ( > 2OD_*C*_). Notably, HQ-1 produced 5.433-fold more biofilm biomass than HQT-2, highlighting its superior potential for ecological persistence and root colonization.

According to Supplementary Figure 2B, root colonization by the two biocontrol strains in *A. membranaceus* increased during the early stages following inoculation. The bacterial populations of GFP-HQ-1 and GFP-HQT-2 peaked at around 8- and 12-days post-inoculation (dpi), respectively, and remained relatively stable thereafter, indicating successful establishment and sustained persistence of the two strains in *A. membranaceus* root tissues.

**FIGURE 6:**
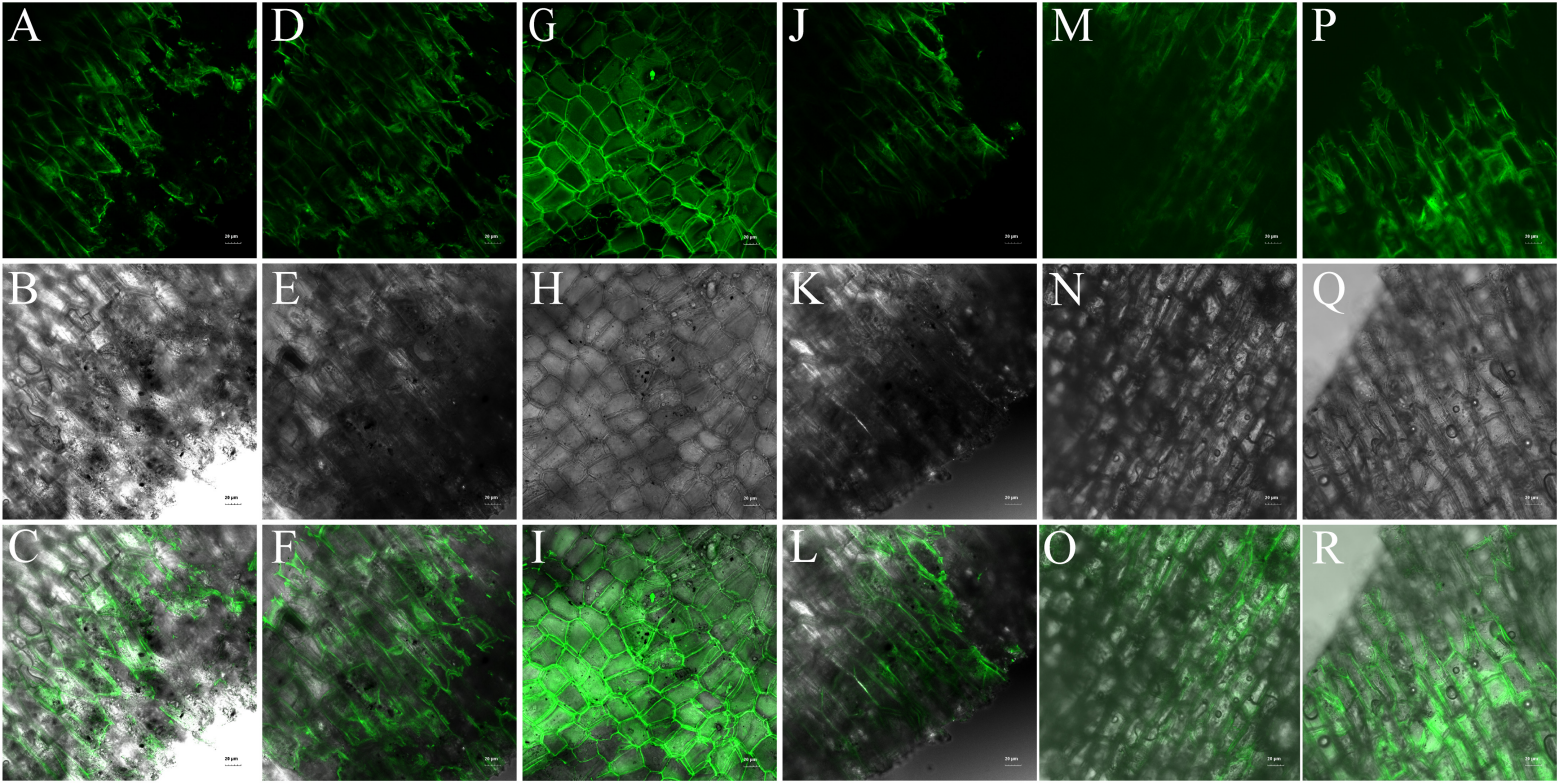
Colonization efficacy of GFP-biocontrol bacterium in *A. membranaceus* roots as visualized by laser confocal microscopy at different time points after inoculation. **(A–C)** The colonization status of GFP-HQ-1 (4 days). **(D–F)** The colonization status of GFP-HQ-1 (12 days). **(G–I)** The colonization status of GFP-HQ-1 (20 days). **(J–L)** The colonization status of GFP-HQT-2 (4 days). **(M–O)** The colonization status of GFP-HQT-2 (12 days). **(P–R)** The colonization status of GFP-HQT-2 (20 days).

Confocal microscopy observations revealed strong fluorescence signals in root tissues at 4 dpi for GFP-HQ-1 and at 12 dpi for GFP-HQT-2, and the signals remained detectable at 20 dpi, further confirming stable colonization by both strains (Figure 6).

### 3.6 Antifungal activity of biocontrol bacterial filtrates: mycelial inhibition and hyphal destruction

**FIGURE 7:**
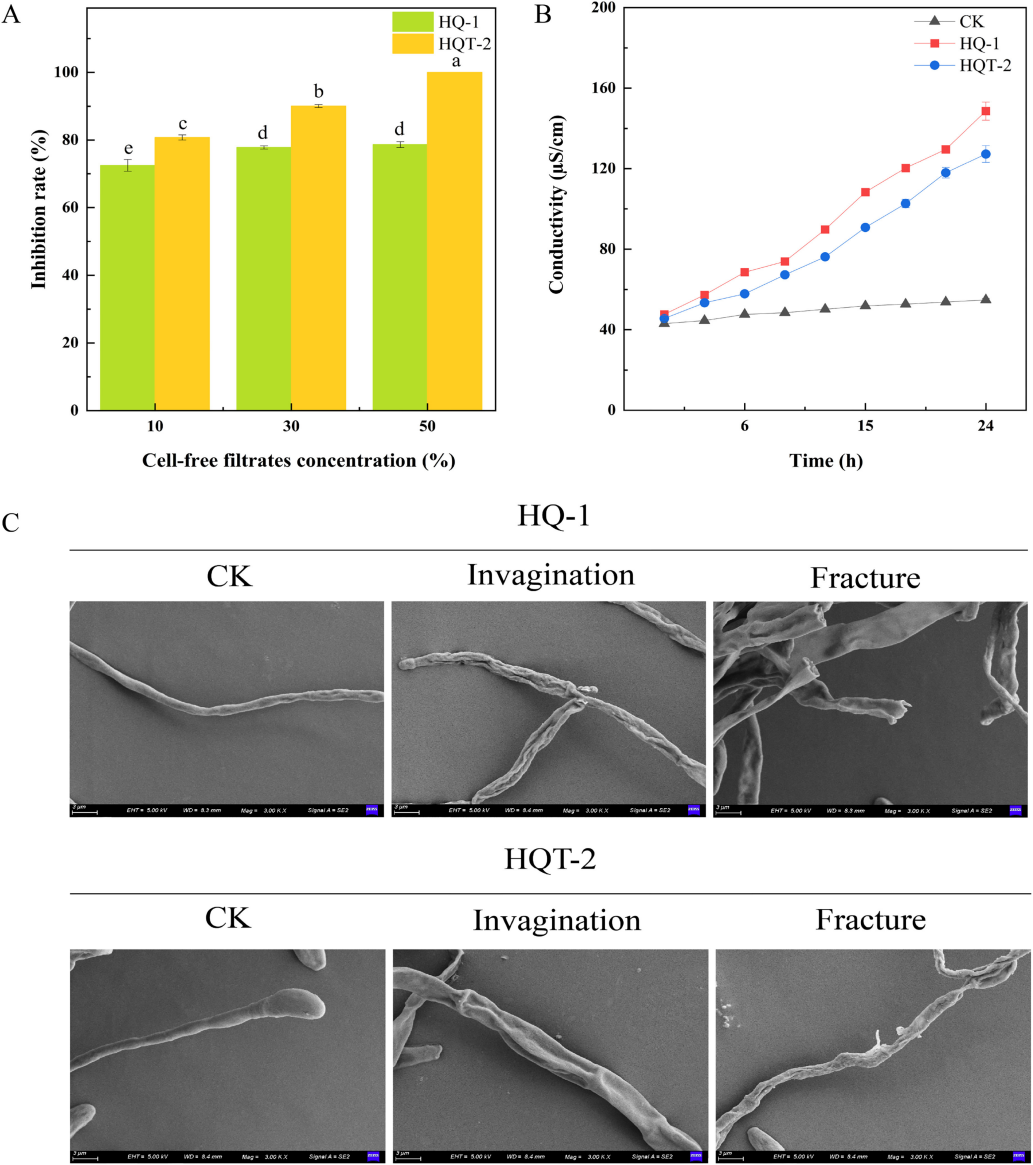
**(A)** Plate hyphae inhibition rate of the cell-free filtrate against GF-3. **(B)** Effects of cell-free filtrates from the two biocontrol bacteria on the conductivity of GF-3 hyphae. **(C)** SEM observation of hyphae damage. Letters a–e above the bars indicate significant differences (*p* < 0.05) according to Tukey’s HSD test. Values with different letters are significantly different, while values with the same letter are not significantly different.

Antifungal assays using cell-free filtrates showed that both significantly inhibited GF-3 growth (Supplementary Figure 3). The filtrate of *P. polymyxa* HQ-1 inhibited growth by 72.54, 77.84, and 78.69% at concentrations of 10, 30, and 50%, respectively. In contrast, *P. terrae* HQT-2 filtrate exhibited significantly stronger inhibition, achieving 80.78% at 10%, 90.08% at 30%, and complete inhibition (100%) at 50% concentration (Tukey’s HSD, *p* < 0.05; Figure 7A).

In the mycelial resuscitation assay, the pathogenic mycelia treated with 50% HQT-2 cell-free filtrate could normally regrow on fresh PDA medium (Supplementary Figure 4). The results confirmed that the inhibitory effect of HQT-2 filtrate was fungistatic rather than fungicidal, suppressing mycelial growth without causing irreversible fungal cell death. The experiment was carried out in triplicate.

Scanning electron microscopy revealed that untreated GF-3 hyphae exhibited a smooth, plump surface, whereas they showed pronounced damage after treatment with strains HQ-1 and HQT-2. Both treatments induced severe morphological alterations, including hyphae shrinkage, invagination, and fracture (Figure 7C). In addition, the conductivity assay revealed a progressive increase in extracellular conductivity in the treatment group exposed to strains HQ-1 and HQT-2, while the control group exhibited only a slight rise (Figure 7B).

### 3.7 Pot experiment on biocontrol efficacy against *A. membranaceus* root rot

**FIGURE 8:**
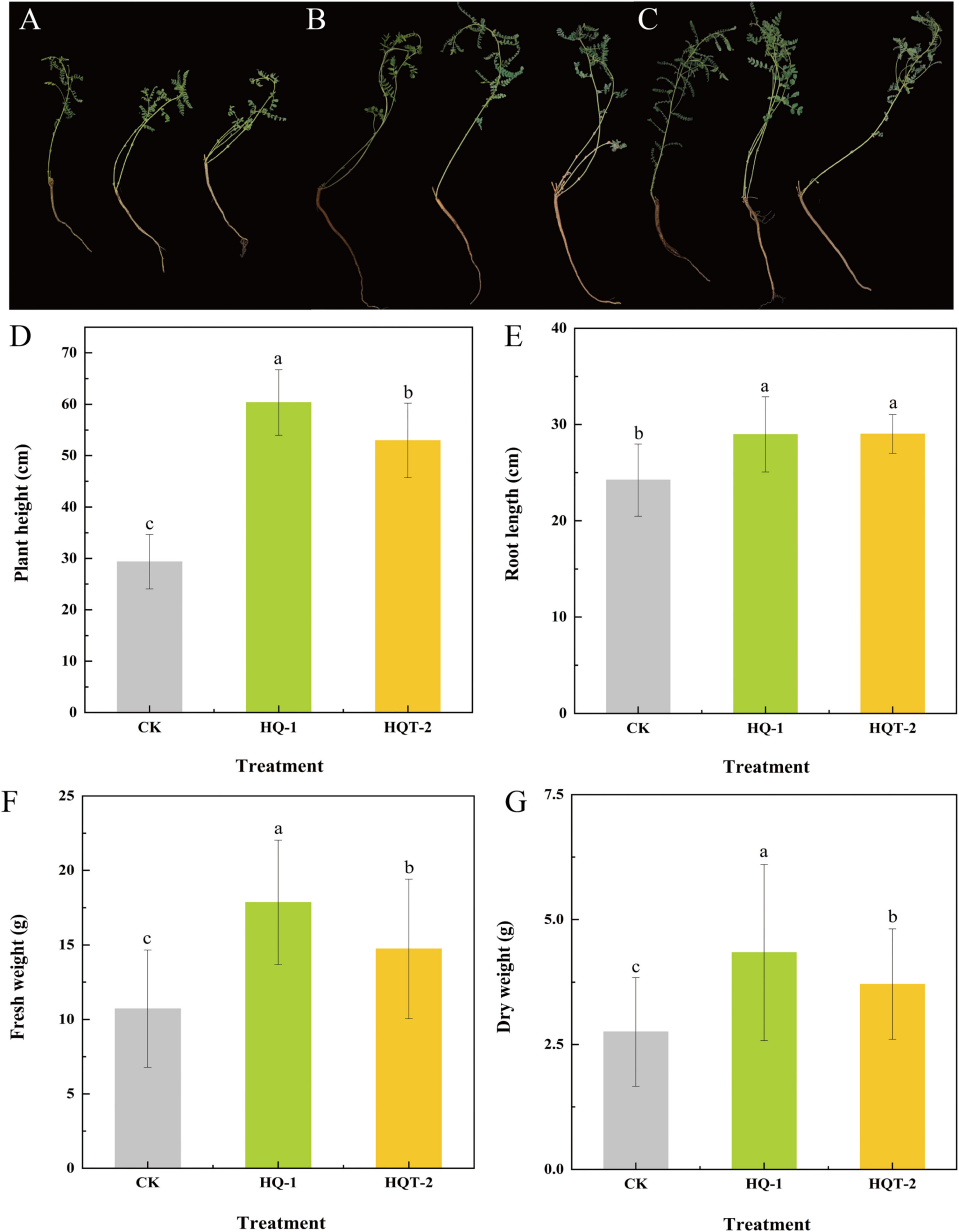
Effects of the two biocontrol bacteria on physiological indices of *A. membranaceus* in the pot experiment. **(A)** CK group of *A. membranaceus*. **(B)** HQ-1 group of *A. membranaceus*. **(C)** HQT-2 group of *A. membranaceus*. **(D)** Plant height. **(E)** Root length. **(F)** Fresh weight. **(G)** Dry weight. Letters a–c above the bars indicate significant differences (*p* < 0.05) according to Tukey’s HSD test. Values with different letters are significantly different, while values with the same letter are not significantly different.

After treatment with HQ-1 and HQT-2, the growth of *A. membranaceus* was significantly promoted (Figure 8). Compared with the CK, HQ-1 showed the most significant growth-promoting effect, with increases of 105.597, 19.546, 66.471, and 57.740% in plant height, root length, fresh weight, and dry weight, respectively. Similarly, HQT-2 also significantly improved plant growth, with corresponding increases of 80.433, 19.868, 37.436, and 34.738%.

For HQ-1, the therapeutic treatment (GF-3 + HQ-1) resulted in a disease index of 4.127 and a control efficacy of 59.690%. In contrast, the preventive treatment (HQ-1 + GF-1) decreased the disease index to 1.429, with a control efficacy of 86.046%. Similarly, for HQT-2, the therapeutic treatment (GF-3 + HQT-2) produced a disease index of 4.444 and a control efficacy of 56.590%, while the preventive treatment (HQT-2 + GF-3) reduced the disease index to 1.984, with a control efficacy of 80.619% (Table 2). Overall, both biocontrol strains showed satisfactory control efficacy against *Astragalus* root rot. Preventive treatments exhibited significantly better disease control effects than therapeutic treatments, indicating a greater potential for disease prevention and management.

**TABLE 2:** Pot experiment on *A. membranaceus* root rot with the two biocontrol bacteria: disease index and control efficacy.

Treatment	MDR^X^	$\bar{\text{R}}$ijy	$\hat{P}$ _ij_	Disease index	Control effect (%)	95% CI for $\hat{P}$_ij_	
						Upper	Lower
CK	0.033	12.467	0.191	0.079 ± 0.14	–	0.215	0.167
GF-3	4.300	53.733	1.026	10.238 ± 0.24	–	0.940	0.818
HQ-1+GF-3	0.600	22.917	0.365	1.429 ± 0.24	86.046	0.488	0.243
GF-3+HQ-1	1.733	33.900	0.548	4.127 ± 0.50	59.690	0.722	0.374
HQT-2+GF-3	0.833	24.867	0.340	1.984 ± 0.50	80.619	0.549	0.247
GF-3+HQT-2	1.867	35.117	0.569	4.444 ± 0.36	56.590	0.748	0.389

Values are presented as mean ± standard error of the mean (SEM) calculated from three independent experiments (*n* = 3). In each independent experiment, each treatment consisted of 10 individual plants. Disease incidence (DI) and disease severity index (DSI) were calculated at the experiment level and subsequently averaged across independent experiments. MDRx, $\bar{\text{R}}$ijy, and $\hat{\text{P}}$_ij_ represent the mean disease rating, mean rank, and relative treatment effect, respectively, with 95% confidence intervals shown for $\hat{\text{P}}$_ij_. Different lowercase letters within the same column indicate statistically significant differences among treatments according to Tukey’s HSD test (*p* < 0.05).

### 3.8 Effects of biocontrol bacteria on plant defense-related enzyme activities

**FIGURE 9:**
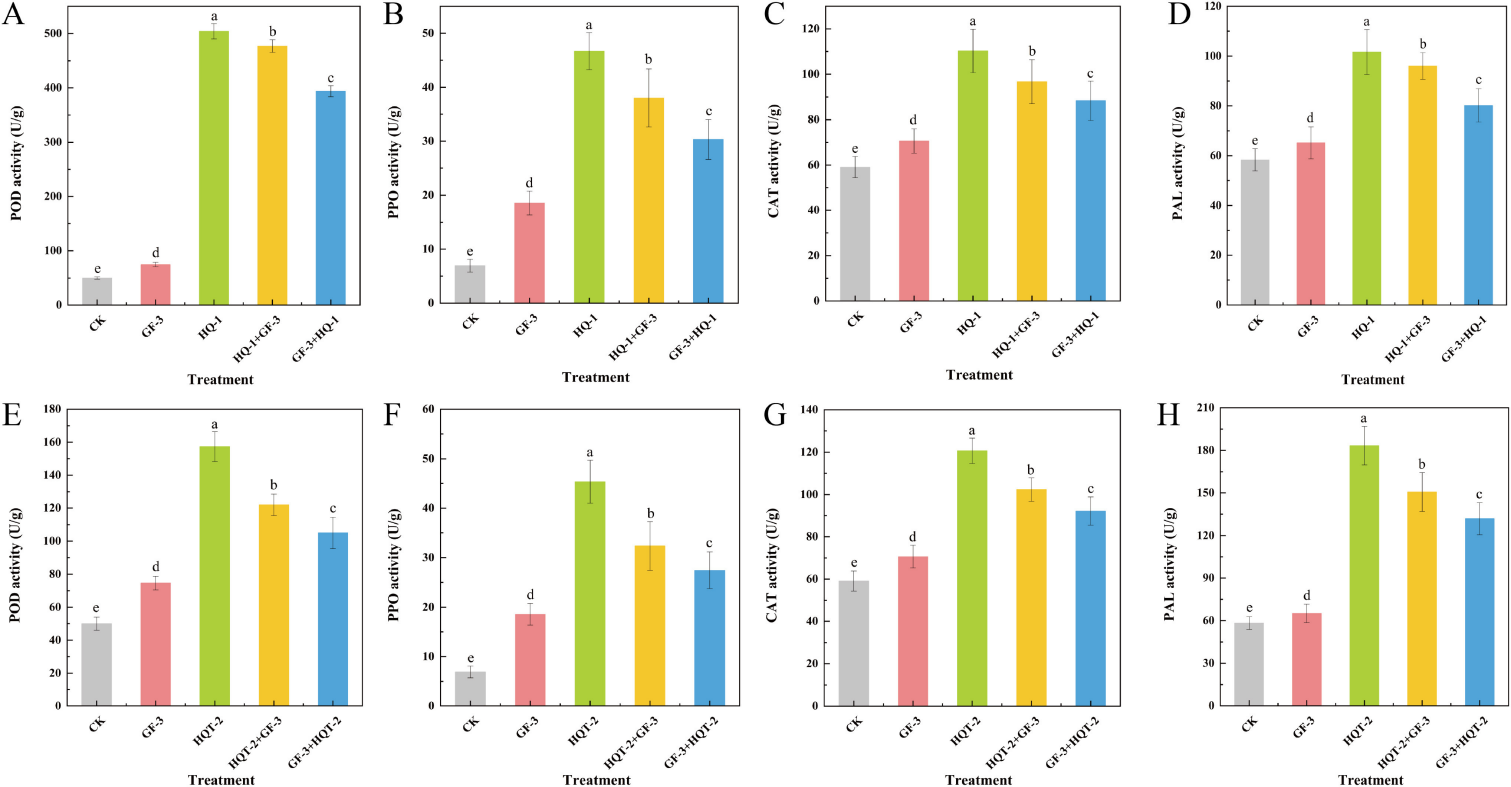
Changes in resistance-related enzyme activities in *A. membranaceus* induced by the growth-promotion, prevention, and treatment experiments with HQ-1 and HQT-2. **(A–D)** HQ-1 group; **(E–H)** HQT-2 group. Letters a–e above the bars indicate significant differences (*p* < 0.05) according to Tukey’s HSD test. Values with different letters are significantly different, while values with the same letter are not significantly different.

Plant defense enzymes induced by biocontrol bacteria play a crucial role in biological control. Inoculation with the biocontrol strains HQ-1 or HQT-2 significantly increased the expression of key defense-related enzymes, including peroxidase (POD), polyphenol oxidase (PPO), catalase (CAT), and phenylalanine ammonia-lyase (PAL) in *A. membranaceus* roots, relative to the control (CK) or the GF-3 treatment (Figure 9). Notably, HQ-1 induced the most pronounced increase, with increases of 10.08-fold for POD, 6.731-fold for PPO, 1.865-fold for CAT, and 1.743-fold for PAL. HQT-2 also significantly increased these enzymes, resulting in increases of 3.16-fold for POD, 6.538-fold for PPO, 2.035-fold for CAT, and 3.143-fold for PAL relative to CK (Tukey’s HSD, *p* < 0.05). These results indicate that both strains effectively prime the host defense system, thereby enhancing its capacity to resist stress.

### 3.9 Induction of endogenous nutrient accumulation in *A. membranaceus* by biocontrol bacteria

**FIGURE 10:**
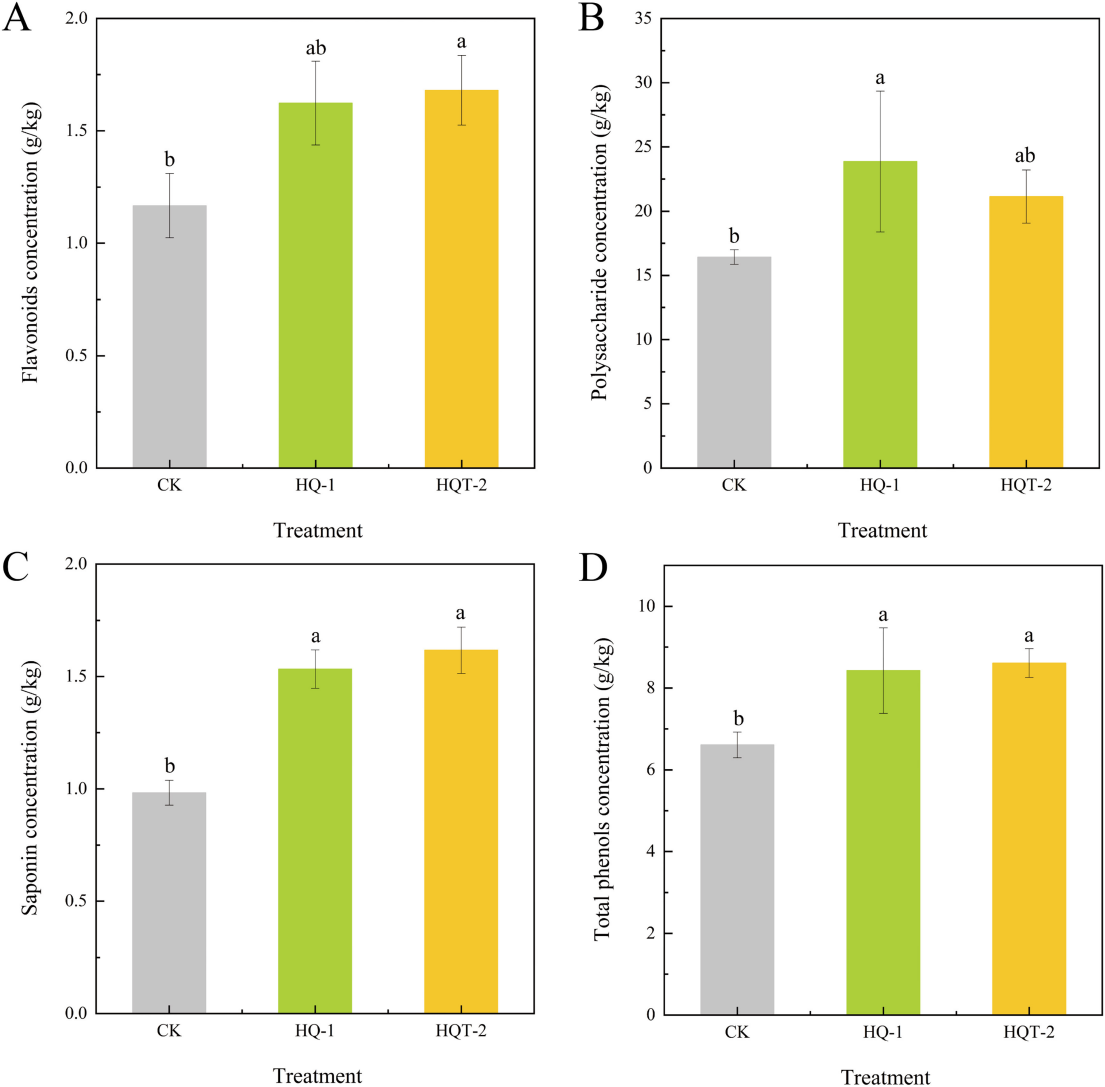
Changes in Endogenous Nutrient Content of *A. membranaceus* Under Biocontrol Bacteria Treatment. **(A)** Flavonoids concentration. **(B)** Polysaccharide concentration. **(C)** Saponin concentration. **(D)** Total phenols concentration. Letters a, b above the bars indicate significant differences (*p* < 0.05) according to Tukey’s HSD test. Values with different letters are significantly different, while values with the same letter are not significantly different.

Treatment with both biocontrol strains enhanced the accumulation of the four major endogenous nutrients in *A. membranaceus*. Compared with the CK group, HQ-1 treatment increased the contents of flavonoids, polysaccharides, saponins, and total phenols by 39.075, 45.214, 55.951, and 27.489%, respectively. Meanwhile, HQT-2 treatment increased their by 43.959, 28.601, 64.496, and 30.257%, respectively (Tukey’s HSD, p < 0.05; Figure 10).

#### 3.9.1 Effects of biocontrol bacteria on soil microbial community diversity

According to Supplementary Figure 5, the application of biocontrol bacteria significantly altered both alpha and beta diversity in soil microbial communities, reshaping soil microbiomes. For alpha diversity, compared with the CK, treatment with HQ-1 significantly decreased the Chao1, ACE, and Shannon indices while increasing the Simpson index, indicating a marked reduction in microbial richness and diversity. In contrast, HQT-2 treatment restored community richness (Chao1, ACE) to intermediate levels, and significantly increased the Shannon index above CK (*p* < 0.05), while also elevating the Simpson index, suggesting a more active role in maintaining community diversity. Beta-diversity analysis revealed clear treatment-specific clustering: PCA showed that PC1 and PC2 explained 85.65 and 12.18% of the total variation, respectively, accounting for 97.83% of the variance. NMDS analysis (stress = 0.0011) further confirmed the distinct separation between groups, demonstrating that biocontrol treatments drove directional shifts in soil microbial community structure.

#### 3.9.2 Impacts on soil microbial community structure

At the phylum rank, both HQ-1 and HQT-2 treatments significantly reduced the relative abundance of *Proteobacteria* (by 13.3 and 32.3%, respectively) and *Bacteroidetes* (by 6.3 and 7.9%, respectively). Conversely, *Actinobacteria* abundance markedly increased under both treatments (by 12.0% in HQ-1 and 37.9% in HQT-2). *Acidobacteria* and *Gemmatimonadetes* abundances showed mixed responses: slight increases in HQ-1 (1.6 and 0.9%, respectively) but minor decreases in HQT-2 (0.6 and 2.1%, respectively). At the genus level, the inoculants increased the relative abundance of several beneficial genera, including *Nocardioides*, *Bradyrhizobium*, *Marmoricola*, *Arthrobacter*, *Gaiella*, *Kribbella*, *Phycicoccus*, among others. Treatment-specific increases were observed for *Lysobacter* and *Pseudolabrys* in HQ-1, and for *Massilia* in HQT-2. Concurrently, both treatments decreased the abundance of genera such as *Sphingomonas*, *Tsuneonella*, *Flavisolibacter*, *Sphingosinicella*, and *Mesorhizobium*. Overall, the biocontrol agents significantly altered the bacterial community composition, leading to the enrichment of beneficial bacteria and reductions in the relative abundance of certain other genera.

**FIGURE 11:**
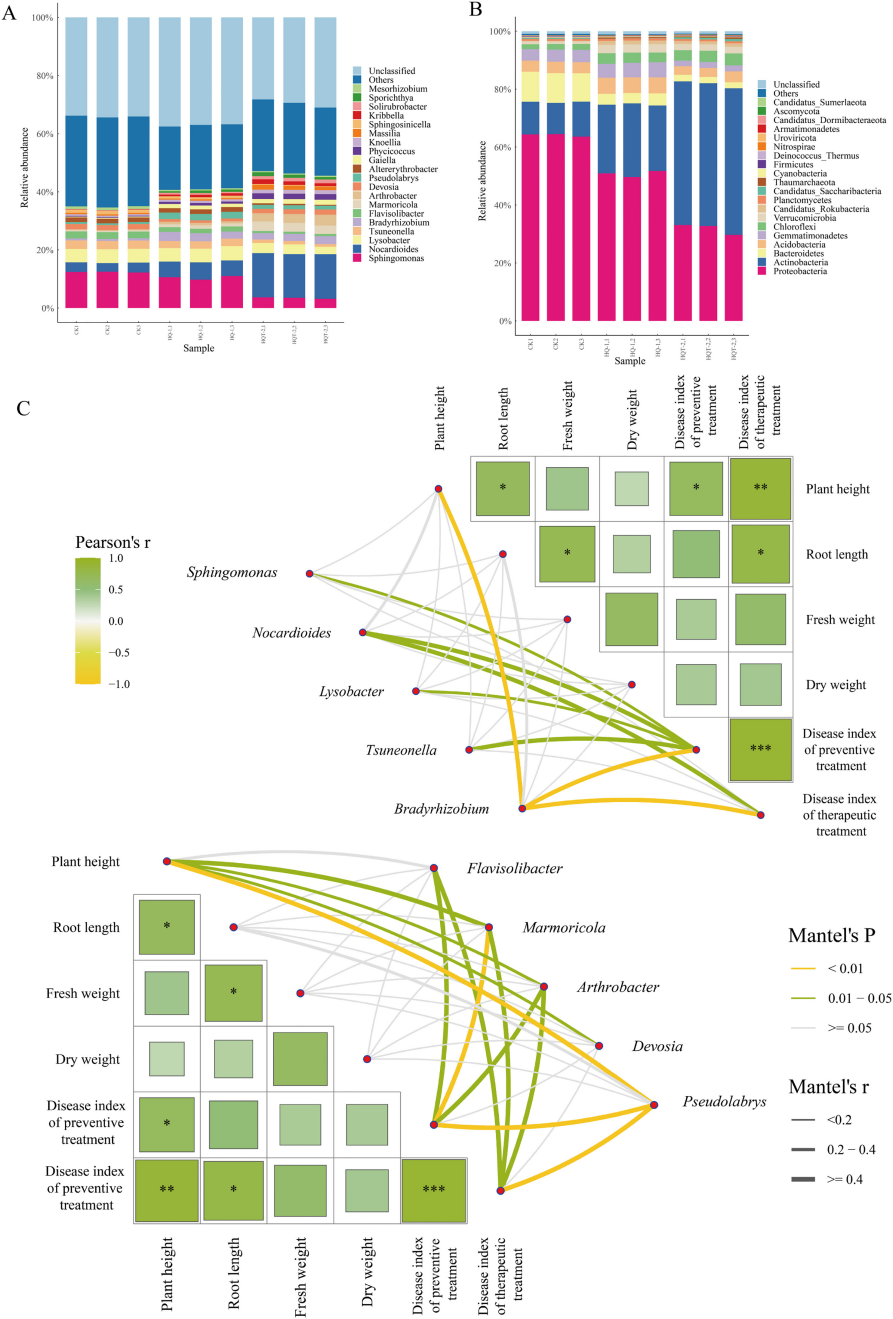
Composition and structure of the soil microbial community. **(A)** Phylum level. **(B)** Genus level. **(C)** Correlation analysis between soil microbial community composition and growth-promoting traits as well as biocontrol efficacy in pot experiments of *A. membranaceus*. *p < 0.05, **p < 0.01, ***p < 0.001.

Correlation analysis showed that microbial communities mediated by HQ-1 were significantly correlated with root elongation, while those by HQT-2 were associated with plant height, fresh weight and dry matter accumulation. Beneficial genera *Nocardioides*, *Arthrobacter* and *Devosia* were positively correlated with growth-promoting traits and negatively correlated with disease indices. These results suggest that the shifts in rhizosphere microbiome structure induced by biocontrol strains HQ-1 and HQT-2 are closely associated with improved plant growth and reduced disease severity, supporting their combined roles in promoting plant performance and alleviating root rot damage (Figure 11). Notably, since metagenomic samples were not collected from pathogen-challenged groups, the observed microbiome changes are not taken as direct evidence for root rot suppression, but rather as potential microbial correlates of the disease-alleviating phenotype observed in our biocontrol experiments.

#### 3.9.3 Differences in the function of soil microbial communities

LEfSe analysis (LDA score > 4, *p* < 0.05) identified distinct microbial biomarkers with significant differences across treatment groups. In the CK group, *Proteobacteria* and *Bacteroidetes* were the dominant taxa, including *Xanthomonadaceae*, a family of broad-spectrum plant pathogens. The HQ-1 treatment enriched biomarkers within the phylum *Gemmatimonadetes*, while the HQT-2 treatment specifically enriched taxa from *Actinobacteria*, notably *Nocardioides* and related taxa, all of which are soil-beneficial bacteria (Supplementary Figure 6). These results indicate that the biocontrol strains HQ-1 and HQT-2 remodel the soil microbiome in a directionally consistent manner, enriching beneficial taxa and suppressing disease-associated microbes, thereby establishing a community profile conducive to plant growth and disease control.

**FIGURE 12:**
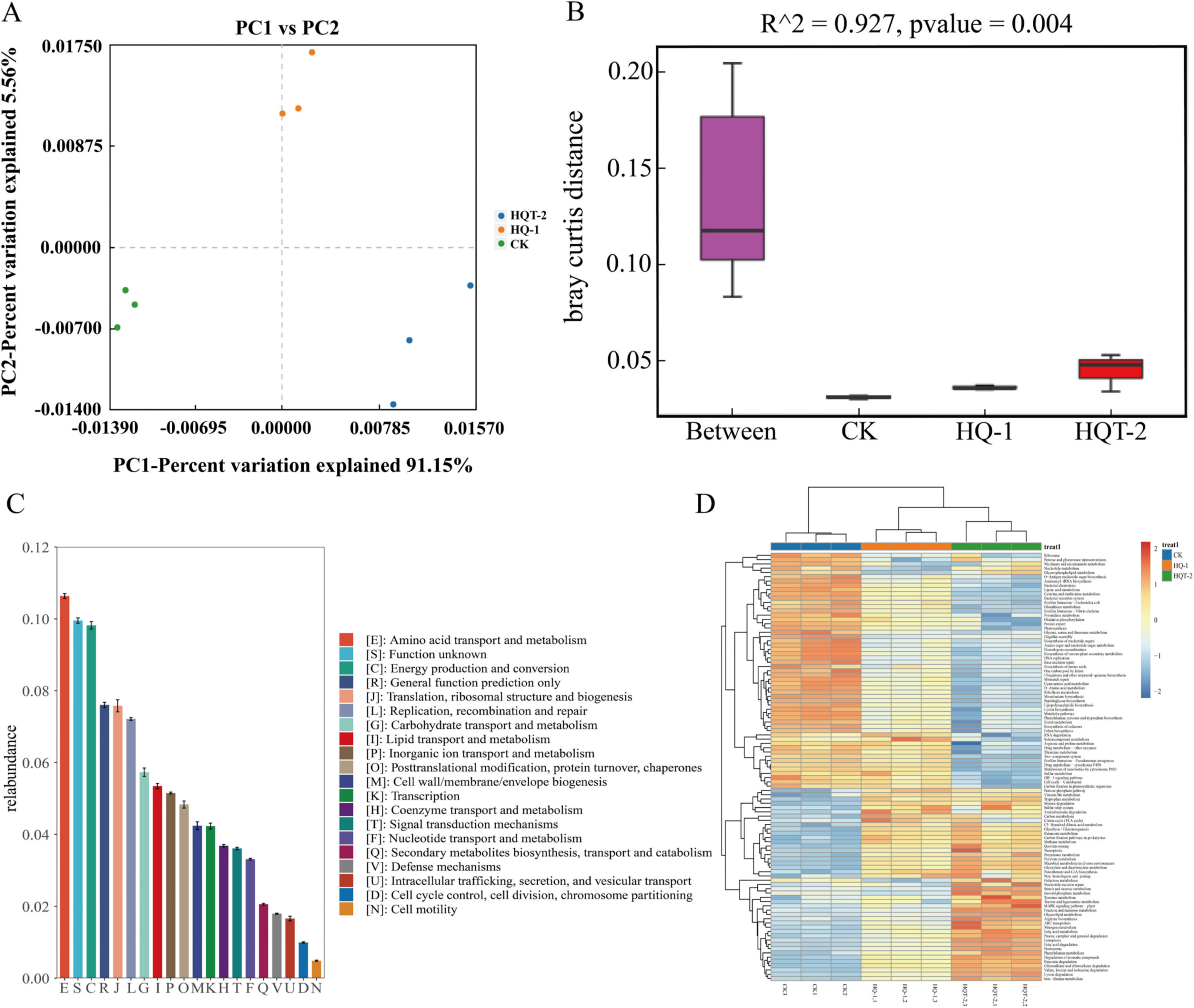
**(A)** PCA of functional genes. **(B)** PERMANOVA analysis of functional genes. **(C)** Microbial community functional annotation based on the KEGG database level 1 and 2. **(D)** KEGG pathway level 3.

PCA analysis showed that PC1 explained 91.15% of the variance, PC2 explained 5.56%, and the cumulative variance contribution was 96.71%, which could effectively reflect differences in functional genes. PERMANOVA based on the Bray-Curtis distance showed that the treatments had a highly significant effect on the structure of functional gene communities (*R*^2^ = 0.927, *F* = 38.33, *p* = 0.004). Among them, the treatment factor accounted for 92.7% of the variation in functional gene communities, indicating that different treatments showed extremely strong specificity and significance in regulating the composition of functional genes. KEGG revealed that both inoculants up-regulated pentose-phosphate, glycolysis, TCA, tryptophan and vitamin-B6 metabolism, as well as aromatic-compound degradation, quorum sensing, and ABC-transporter pathways linked to plant growth and defense (Figure 12).

## 4 Discussion

In the present study, two bacterial strains, *P. polymyxa* HQ-1 and P. terrae HQT-2, exhibited antagonistic activity. Both strains not only suppressed *F. solani* GF-3 (the pathogen of *A. membranaceus* root rot), but also exhibited broad-spectrum antagonism against multiple soil-borne pathogens, indicating their considerable potential for field application. Cell-free filtrates of HQ-1 and HQT-2 caused marked hyphal deformation, shrinkage, and lysis of *F. solani* GF-3, concomitant with electrolyte leakage. Previous reports have demonstrated that *Paenibacillus* spp. secretes cellulases and β-1,3-glucanases (Gong et al., 2025) that degrade fungal cell walls, leading to structural collapse and the extrusion of intracellular proteins and nucleic acids. Our results are consistent with these findings. The plate inhibition assay using cell-free filtrates showed that HQT-2 exhibited a stronger antagonistic effect, especially at 50%. HQ-1 and HQT-2 colonized *A. membranaceus* roots, forming cell aggregates or microcolonies within root tissues and thereby competing with pathogens for intra-root niche occupation. Moreover, the colonization ability of HQ-1 was superior to that of HQT-2, consistent with the colonization potential inferred from the biofilm formation assay, as the biofilm-forming capacity of strains is often positively correlated with their colonization ability (Liu et al., 2025). This trait may explain why HQ-1 showed stronger disease control efficacy in the pot experiment despite similar antagonistic activities in dual-culture tests. Earlier studies have established that the efficient rhizosphere-colonizing capability of biocontrol bacteria is closely linked to motility and biofilm formation (Guo et al., 2020), the latter being a pivotal mechanism for forming functional microbial consortia on plant surfaces (Xia et al., 2020). Biofilm formation enhances microbial tolerance to environmental stresses, maintains bacterial populations at high densities in an aggregated state, and facilitates firm colonization of the root system, enabling the biocontrol population to effectively occupy infection sites, compete for nutrients and space, and synergize with host defense responses, ultimately reducing disease incidence (Wang Q. et al., 2022). Following root colonization, *Paenibacillus* spp. can counteract a variety of soil-borne diseases not only through secreted lytic enzymes but also via an array of antimicrobial metabolites.

Pot experiments showed that both HQ-1 and HQT-2 markedly promoted the growth of *A. membranaceus*. Further research has revealed that both strains synthesized indole-3-acetic acid (IAA), a pivotal phytohormone that modulates plant growth, development, and stress responses (Padda et al., 2016; Shao et al., 2025; Idris et al., 2007). Additionally, both strains secreted siderophores—low-molecular-mass, high-affinity iron-chelators that scavenge Fe^3+^ from the soil, form ferric–siderophore complexes, and deliver iron to the plant, thereby alleviating iron limitation and enhancing plant growth (Deng et al., 2023). Members of the genus *Paenibacillus* are known for synthesizing siderophores and other iron chelators that improve plant performance under iron-deficient conditions. Both strains exhibit documented plant-growth-promoting (PGP) traits, including phosphorus solubilization, potassium mobilization, and biological nitrogen fixation. More than 95% of phosphate-solubilizing bacteria convert insoluble phosphates into plant-available forms, thereby enhancing phosphorus uptake (Yang et al., 2018). Whereas diazotrophic bacteria convert molecular nitrogen into plant-available ammonium via the nitrogenase system (Li J. et al., 2025) and potassium-mobilizing bacteria secrete organic acids that release soluble K^+^ into the rhizosphere (Han et al., 2022). Thus, the notable PGP attributes of HQ-1 and HQT-2 enrich soil nutrients and enhance their bioavailability, explaining the superior growth-promoting effects observed in the pot experiments.

*Paenibacillus* spp. can prime plant immunity even in the absence of pathogens by coordinately activating defense-related enzymes (PAL, POD, CAT, and PPO). Both viable and heat-killed cells of *P. polymyxa* KN-03 elicit induced resistance in citrus seedlings (Yang, 2022), strain JNJX-2 elevates defensive enzyme activities in cotton (Jia, 2018), and *P. terrae* NK3-4 induces maize resistance by up-regulating PAL activity and CAT gene expression (Yan, 2020). In the present study, colonization by *Paenibacillus* strains consistently elevated the activities of these defense enzymes even in the absence of pathogens. Consequently, HQ-1 and HQT-2 can serve as compatible co-inoculants to potentiate induced systemic resistance (ISR) in *A. membranaceus* under non-infectious conditions. In addition, the application of biocontrol bacteria can promote the accumulation of endogenous nutrients in *A. membranaceus* and improve its quality and nutritional value. Consistent with previous studies, *P. polymyxa* can increase the total saponin content of ginseng by 6.0–12.1% (Li Y. et al., 2025) and increase the content of tea polyphenols in tea leaves (Wang and Li, 2022).

The introduction of the biocontrol strains markedly reshaped the soil bacterial community. At the phylum level, *Proteobacteria*, *Actinobacteria*, *Bacteroidota*, and *Chloroflexi* emerged as the most dominant phyla in farmland soils, corroborating previous studies (Li, 2024). Although the overall composition remained unchanged, the relative abundances of these phyla were significantly altered and the proportions of beneficial taxa were increased. For example, *Bradyrhizobium* can establish symbiotic nitrogen fixation, thereby reducing chemical fertilizer input and increasing crop yields (Zhang et al., 2023), beyond this symbiotic N2-fixation capacity, these rhizobia are also capable of forming cell aggregates or micro-colonies, which may act as a specific niche to compete with pathogenic microorganisms for the colonization of intra-root habitats (Kebede, 2021). *Arthrobacter* can act as a biocontrol bacterium, significantly suppressing the pathogen that causes tomato bacterial wilt (Huang et al., 2013). Treatment with strain HQ-1 notably increased the relative abundance of *Lysobacter*, which colonizes roots via pili and induces the expression of PAL, POD, and PR proteins, leading to a lower disease index (Ren, 2020). We infer that HQ-1 exhibited superior biocontrol efficacy in the *A. membranaceus* assay, with *Lysobacter* playing a crucial role.

LEfSe analysis identified 42 differential bacterial biomarkers between the CK group and the HQ-1/HQT-2 groups, confirming that the biocontrol strains can effectively reshape the rhizosphere micro-ecology of *A. membranaceus*. This reshaping effect is closely linked to the previously reported antagonistic and plant-growth-promoting (PGP) mechanisms of the two strains. Functionally, the 15 CK-enriched biomarkers clustered within *Proteobacteria*, with the core family *Xanthomonadaceae*—containing multiple phytopathogens—indicating that their relative enrichment is associated with elevated disease risk (Chuang et al., 2025). The nine HQ-1 biomarkers belonged to *Gemmatimonadetes*, a phylum that mainly drives carbon and nitrogen cycling; its proliferation suppresses pathogen colonization through niche competition and synergistically enhances PGP performance (Oshiki et al., 2022). The 18 HQT-2 biomarkers were dominated by *Actinobacteria* (*Nocardioides* and *Chitinophagales*). *Nocardioides* is a classic biocontrol genus, while *Chitinophagales* can degrade chitin; together, they reinforce rhizosphere antimicrobial activity and inhibit pathogen proliferation by disrupting fungal cell walls, providing a microbiological supplement to the disease-suppressive function of HQT-2. Collectively, HQ-1 and HQT-2 achieve directional rhizosphere manipulation by enriching nutrient-cycling or biocontrol consortia while simultaneously reducing pathogen populations, integrating these changes with the strains’ enzymatic antagonism and hormone-mediated PGP to form a unified growth-promotion-disease -suppression system.

From the KEGG functional annotation results, metabolic pathways dominated the level 1 functions. The upregulation of core carbon metabolic pathways enhances the efficiency of biocontrol bacteria in utilizing rhizospheric carbon sources, and their metabolites can directly provide nutrients and growth signals for plants (Wang et al., 2021), which is consistent with previous research conclusions that biocontrol bacteria such as *Arthrobacter* and *Nocardioides* possess both growth-promoting and disease-suppressive functions (Zhang J. et al., 2024). Meanwhile, the activation of metabolic pathways such as vitamin B6 metabolism further provides coenzyme-like regulatory substances for plant growth (Elisa et al., 2017), supporting the promoting effect of biocontrol bacteria on plant biomass accumulation. Additionally, the upregulation of aromatic compound degradation pathways indicates that biocontrol bacteria can achieve direct antagonism by degrading pathogen toxins and synthesizing antimicrobial secondary metabolites. The activation of quorum-sensing pathways helps biocontrol bacteria synergistically secrete antimicrobial substances (Meng et al., 2021) while interfering with pathogen signaling to enhance competitive advantages. This provides evidence at the metabolic pathway level for deciphering the disease control mechanism of biocontrol bacteria (Zheng et al., 2025). Ultimately, this forms a biocontrol bacterium-microbial community-plant interaction network, collectively supporting their integrated effects of promoting plant growth and suppressing disease.

Existing studies on *P. terrae* have mainly focused on diseases of other crops. For example, Smith et al. only conducted an *in vitro* antagonism test of *P. terrae* B6a against *Fusarium proliferatum* on maize seeds, without carrying out pot or field experiments for biological control of root rot and stalk rot caused by this pathogen at the adult maize stage, and lacked comprehensive research on colonization dynamics, disease severity, and comprehensive biocontrol efficacy (Smith et al., 2025). Liu et al. investigated only the growth-promoting effect of *P. terrae* NK3-4 on rice (Liu et al., 2023). In contrast, this study systematically evaluated the biocontrol efficacy of *P. terrae* against root rot of medicinal plants, further verified root colonization via GFP tagging, and performed complete *in vitro* and *in vivo* biocontrol assays. These results fill the research gap on *P. terrae* as a biocontrol agent for medicinal plants and provide more comprehensive and systematic evidence of its application potential.

Meanwhile, *P. polymyxa* has been shown to degrade various organic compounds, including pentachloronitrobenzene (Tian, 2023). It can improve the rhizosphere microenvironment, eliminate stress factors, and thus enhance the overall disease control efficacy. Subsequent research will focus on the degradation of harmful compounds by HQ-1 and HQT-2, futher explore their biocontrol functions, investigate the molecular mechanisms underlying their degradation capacity, and provide a stronger research basis for biological and green disease control.

## 5 Conclusion

In this study, two effective biocontrol strains, *P. polymyxa* HQ-1 and *P. terrae* HQT-2, were isolated from the rhizosphere of *A. membranaceus*. They demonstrated high biocontrol efficiency (80.62–86.05%) against *F. solani* in pot trials and significantly promoted plant growth. With stable root colonization, these strains exert dual biocontrol functions: directly antagonizing pathogens through antimicrobial secretion and indirectly stimulating host resistance by enhancing of defense enzyme activity. Furthermore, they reshaped the rhizosphere microbiome by enriching beneficial taxa, suppressing pathogens, and upregulating defense- and metabolism-related genes. Our findings establish HQ-1 and HQT-2 as valuable, multifunctional microbial resources for development of targeted biocontrol agents and biofertilizers for *A. membranaceus* cultivation. Notably, research focused on *P. terrae* remains relatively scarce, with only a few studies investigating its biocontrol potential against plant pathogens. Here, we report a novel strain, HQT-2, that exhibits potent biocontrol activity against relevant pathogens, thereby expanding the application scope of *P. terrae* in plant disease management and advancing this field.

## Data Availability

The datasets presented in this study can be found in online repositories. The data that support the findings of this study are deposited in NCBI with accession numbers PX844720, PX844714 and PRJNA1401705.

## Supplementary material

Supplementary Figure 1 Phylogenetic tree of the two biocontrol bacterial strains. **(A)** HQ-1. **(B)** HQT-2. **(C)** Gram staining of HQ-1. **(D)** Gram staining of HQT-2.

Supplementary Figure 2 **(A)** Biofilm-forming ability. **(B)** Hyphal conductivity.

Supplementary Figure 3 Inhibitory effect of cell-free filtrates from the two biocontrol agents on the pathogen. **(A)** HQ-1. **(B)** HQT-2.

Supplementary Figure 4 Mycelial resuscitation assay. **(A)** Original strain GF-3. **(B)** Resuscitated strain GF-3.

Supplementary Figure 5 Alpha diversity and Beta diversity of the soil microbial community. **(A)** Chao 1. **(B)** Shannon index. **(C)** Simpson index. **(D)** ACE index. **(E)** PCA analysis. **(F)** NMSD analysis.

Supplementary Figure 6 Cladogram of intergroup LEfSe analysis.

## References

[B1] Cao R. L. Wang Q. L. Sun C. Q. Xve Z. Q. Liu Z. Y. Hu P. et al. (2025). Isolation, identification and biological characteristics of *Proteus mirabilis* from Sika Deer. *Microbiol. China* 52 2230–2243. 10.13344/j.microbiol.china.240699

[B2] Cao Y. Zhang Z. H. Ling N. Yuan Y. J. Zheng X. Y. Shen B. et al. (2011). *Bacillus subtilis* SQR 9 can control *Fusarium* wilt in cucumber by colonizing plant roots. *Biol. Fertil. Soils* 47 495–506. 10.1007/s00374-011-0556-2

[B3] Chuang S. C. Dobhal S. Keith L. M. Alvarez A. M. Arif M. (2025). *Xanthomonas* spp. infecting araceae and araliaceae: Taxonomy, phylogeny, and potential virulence mechanisms. *Biology* 14:766. 10.3390/biology14070766 40723327 PMC12292232

[B4] Cui W. Y. He P. J. Munir S. He P. B. Li X. Y. Li Y. M. et al. (2019). Efficacy of plant growth promoting bacteria B9601-Y2 for biocontrol of southern corn leaf blight. *Biol. Control* 139:104080. 10.1016/j.biocontrol.2019.104080

[B5] Deng S. K. Lei F. J. Long Y. P. Zhang H. R. Jiang Y. X. Zhang A. H. (2023). Research progress on siderophore-mediated antagonism against plant-pathogenic fungi and plant-growth-promoting effects by bacteria. *Microbiol. China* 50 3198–3210. 10.13344/j.microbiol.china.220964

[B6] Elisa D. A. Boycheva S. Fitzpatrick T. B. (2017). The pseudoenzyme PDX1.2 sustains vitamin B_6_ biosynthesis as a function of heat stress. *Plant Physiol.* 174 2098–2112. 10.1104/pp.17.00531 28550206 PMC5543961

[B7] Fan L. S. Huang Z. B. Jin X. J. Lin H. L. Liu X. Meng J. J. et al. (2025). Effects of three biological bacteria on the control of *Astragalus* root rot and plant growth. *Chin. J. Pestic. Sci.* 27 160–170. 10.16801/j.issn.1008-7303.2025.0008

[B8] Gong C. Zhang W. H. Zhang P. Lang D. M. (2025). Screening, identification, and degradation capacity of highly efficient cellulolytic bacteria from saline-alkali soils in northern Henan. *J. Henan Inst. Sci. Technol.* 53 8–14. 10.3969/j.issn.2096-9473.2025.02.002

[B9] Guardado-Fierros B. G. Tuesta-Popolizio D. A. Lorenzo-Santiago M. A. Rodriguez-Campos J. R. Contreras-Ramos S. M. (2024). Comparative study between Salkowski reagent and chromatographic method for auxins quantification from bacterial production. *Front. Plant Sci.* 15:1378079. 10.3389/fpls.2024.1378079 38947947 PMC11212217

[B10] Guo Q. Shi M. D. Chen L. Zhou J. H. Zhang L. X. Li Y. L. et al. (2020). The biocontrol bacterium *Streptomyces pactum* increases *Pseudomonas koreensis* populations in the rhizosphere by enhancing chemotaxis and biofilm formation. *Soil Biol. Biochem.* 144:107755. 10.1016/j.soilbio.2020.107755

[B11] Han M. Zhu X. Y. Chen G. W. Wan X. M. Wang G. (2022). Research progress on potassium-solubilizing bacteria and their potassium-releasing micro-mechanisms. *Acta Hortic. Sin.* 59 334–348. 10.11766/trxb202009190525

[B12] Huang M. Y. Gu W. J. Zhang F. B. Lu Y. S. Xie K. Z. Jiang R. P. (2013). Stability assessment of *Arthrobacter sp*. YB6 cell-free culture filtrate and its control efficacy against tomato bacterial wilt in pot experiments. *Guangdong Agric. Sci.* 40 75–78. 10.16768/j.issn.1004-874x.2013.18.036

[B13] Huang Y. S. Xie X. W. Shi Y. X. Chai A. L. Li L. Li B. J. (2020). Control effect of *Paenibacillus polymyxa* ZF197 against Chinese cabbage stem base rot. *Acta Hortic. Sin* 47 1059–1071. 10.16420/j.issn.0513-353x.2019-0915

[B14] Idris E. E. Iglesias D. J. Talon M. Borriss R. (2007). Tryptophan-Dependent production of Indole-3-Acetic Acid (IAA) affects level of plant growth promotion by *Bacillus amyloliquefaciens* FZB42. *Mol. Plant Microbe Interact.* 20 619–626. 10.1094/MPMI-20-6-0619 17555270

[B15] Jeffrey J. C. (2016). The *Fusarium solani* species complex: Ubiquitous pathogens of agricultural importance. *Mol. Plant Pathol.* 17 146–158. 10.1111/mpp.12289 26531837 PMC6638333

[B16] Jia S. Q. Liu Y. X. Wang Z. C. Zang J. P. Zhang K. Dong J. G. et al. (2025). Screening and identification of biocontrol strains against maize stalk rot and their field control efficacy. *Microbiol. China* 52 3111–3123. 10.13344/j.microbiol.china.240999

[B17] Jia X. C. (2018). *Screening of Biocontrol Bacteria for Cotton Verticillium Wilt and Study on the Mechanism of Disease Resistanc.* Master’s thesis, China: Shandong Normal University.

[B18] Jiang J. J. Chen A. C. Wei Z. Q. Sun X. M. Xu M. R. Li X. P. et al. (2023). Pathogen identification of *Fusarium* root rot of *Scutellaria baicalensis* and changes in root element contents of diseased plants in Longxi, Gansu. *Acta Prataculturae Sinica* 32 109–121. 10.11686/cyxb2022318

[B19] Kalantari S. Marefat A. Naseri B. Hemmati R. (2018). Improvement of bean yield and *Fusarium* root rot biocontrol using mixtures of *Bacillus*, *Pseudomonas* and *Rhizobium*. *Trop. Plant Pathol.* 43 499–505. 10.1007/s40858-018-0252-y

[B20] Kebede E. (2021). Competency of rhizobial inoculation in sustainable agricultural production and biocontrol of plant diseases. *Front. Sustain. Food Syst.* 5:728014. 10.3389/fsufs.2021.728014

[B21] Khan M. S. Gao J. Chen X. Zhang M. Yang F. Du Y. et al. (2020). Isolation and characterization of plant growth-promoting endophytic bacteria *Paenibacillus polymyxa* SK1 from *Lilium lancifolium*. *Biomed. Res. Int.* 2020:8650957. 10.1155/2020/8650957 32190683 PMC7064867

[B22] Li J. Chen W. Lu Z. Li H. Chi X. Ma X. et al. (2025). Nanoengineered azotobacter *Pseudomonas stutzeri* A1501 for soil ecology restoration and biological nitrogen fixation. *ACS Nano* 19 18143–18155. 10.1021/acsnano.4c15823 40343854

[B23] Li K. M. (2024). *Response of the Microbial Community in the Rhizosphere Soil of Suaeda Salsa Under Petroleum-Contamination Stress.* Master’s thesis, China: Dalian University of Technology, 10.26991/d.cnki.gdllu.2024.003934

[B24] Li R. H. Li H. X. Li C. S. Liu P. J. Wang F. Gu Y. (2022). Effects of microbial inoculants on biomass of continuous cropping *Astragalus membranaceus* var. mongholicus and soil fungal community structure. *J. North. Agric.* 50 50–56. 10.12190/j.issn.2096-1197.2022.06.07

[B25] Li X. J. Yao C. X. Qiu R. Bai J. K. Liu C. Chen Y. G. et al. (2023). Isolation, identification, and evaluation of the biocontrol potential of a *Bacillus velezensis* strain against Tobacco root rot caused by *Fusarium oxysporum*. *J. Appl. Microbiol.* 134:lxac049. 10.1093/jambio/lxac049 36626796

[B26] Li X. P. Qu Y. H. Yang Y. Nong Q. D. Ru R. H. Li C. Q. et al. (2025). Toxicity determination of eight fungicides with different mechanisms of action against two pathogens of *Panax Notoginseng* root rot disease. *China Trop. Agric.* 6 52–58+23.

[B27] Li Y. Sun C. Wu W. C. Bao L. E. Liang Y. J. (2025). Application prospects of microorganisms in improving the quality of panax medicinal plants. *Northern Hortic.* 3, 121–128. 10.11937/bfyy.20242616

[B28] Li Z. L. Liu S. Wang Y. Y. Zhou Y. Liu Q. Yin K. D. (2021). Screening and identification of 5 salt-alkali-tolerant plant-growth-promoting bacteria strains and their growth-promoting effects on *Vigna angularis*. *Microbiol. China* 48 1580–1592. 10.13344/j.microbiol.china.200760

[B29] Lin Y. X. (2025). *Mechanism Exploration of Volatile Substances Produced by Stenotrophomonas sp. B-45 in Controlling Astragalus Membranaceus Root Rot.* Master’s thesis, China: Northwest A&F University, 10.27409/d.cnki.gxbnu.2025.001776

[B30] Liu H. Wang J. Sun H. Han X. Peng Y. Liu J. et al. (2020). Transcriptome profiles reveal the growth-promoting mechanisms of *Paenibacillus polymyxa* YC0136 on Tobacco (*Nicotiana tabacum* L.). *Front. Microbiol.* 11:584174. 10.3389/fmicb.2020.584174 33101258 PMC7546199

[B31] Liu W. Li Z. Liu C. Yu X. Yu W. Li P. (2023). *Paenibacillus terrae* NK3-4 regulates the transcription of growth-related and stress resistance-related genes in rice. *Genome* 66 131–149. 10.1139/gen-2022-0072 36927123

[B32] Liu Y. Li Q. Lan Y. Li Z. Zhao X. Liu Y. et al. (2025). Correlation analysis between biofilm formation of maize symbiotic bacteria and their root surface colonization. *J. Northeast Agric. Sci.* 50 108–115. 10.16423/j.cnki.1003-8701.2025.06.018

[B33] Ma J. X. (2022). *Screening of Biocontrol Bacteria against Fusarium Wilt and Evaluation of Their Control Effects in Watermelon.* Master’s thesis, China: Lanzhou Jiaotong University, 10.27205/d.cnki.gltec.2022.001103

[B34] Meng F. Q. Zhao H. Z. Nie T. Lu F. X. Zhang C. Lu Y. J. et al. (2021). Acetate activates *Lactobacillus bacteriocin* synthesis by controlling quorum sensing. *Appl. Environ. Microbiol.* 87:e00720–21. 10.1128/AEM.00720-21 33893120 PMC8315994

[B35] Mu R. R. (2024). *Biological Control and Mechanism of Angelica Root Rot.* Master’s thesis, China: Lanzhou Jiaotong University, 10.27205/d.cnki.gltec.2024.000057

[B36] Oshiki M. Toyama Y. Suenaga T. Terada A. Kasahara Y. Yamaguchi T. et al. (2022). N_2_O reduction by *Gemmatimonas aurantiaca* and potential involvement of *Gemmatimonadetes Bacteria* in N_2_O reduction in agricultural soils. *Microbes Environ.* 37:ME21090. 10.1264/jsme2.ME21090 35418546 PMC9530729

[B37] Padda K. P. Puri A. Chanway C. P. (2016). Effect of GFP tagging of *Paenibacillus polymyxa* P2b-2R on its ability to promote growth of canola and tomato seedlings. *Biol. Fertil Soils* 52 377–387. 10.1007/s00374-015-1083-3

[B38] Ren S. S. (2020). *Identification and Functional Characterization of the Type VI Secretion System Gene Cluster in Lysobacter enzymogenes OH11.* Master’s thesis, China: Nanjing Agricultural University, 10.27244/d.cnki.gnjnu.2020.000857

[B39] Shang R. Mao J. He S. Q. Li G. Liu C. Z. (2025). Isolation and identification of pathogens causing root rot of *Polygonatum kingianum* in Xishuangbanna. *Trop. Agric. Sci. Technol.* 48 53–57. 10.16005/j.cnki.tast.20250305

[B40] Shao J. Xu Z. Zhang N. Shen Q. Zhang R. (2025). Contribution of indole-3-acetic acid in the plant growth promotion by the rhizospheric strain Bacillus amyloliquefaciens SQR9. *Biol. Fertil. Soils* 51 321–330. 10.1007/s00374-014-0978-8

[B41] Smith E. Daniel A. I. Smith C. Fisher S. Nkomo M. Keyster M. et al. (2025). Exploring *Paenibacillus terrae* B6a as a sustainable biocontrol agent for *Fusarium proliferatum*. *Front. Microbiol.* 3:1547571. 10.3389/fmicb.2025.1547571 40099181 PMC11911495

[B42] Su M. Tang T. Tang W. Long Y. Wang L. Liu M. (2023). *Astragalus* improves intestinal barrier function and immunity by acting on intestinal microbiota to treat T2DM: A research review. *Front. Immunol.* 10:1243834. 10.3389/fimmu.2023.1243834 37638043 PMC10450032

[B43] Tian Y. (2023). *Effects of Symbiotic Bacteria and Trace Elements on the Pesticide Residue Degradation Efficiency of Paenibacillus Polymyxa.* Master’s thesis, China: Jilin Agricultural University, 10.27163/d.cnki.gjlnu.2023.000064

[B44] Wang J. (2014). *Isolation and Identification of Pathogens Causing Root Rot of Panax Notoginseng and Screening of Fungicides for Control.* Master’s thesis, China: Northwest A&F University.

[B45] Wang J. H. Li H. L. (2022). Effects of *Paenibacillus polymyxa* fertilizer on tea plant growth and tea quality. *J. Gansu Sci.* 34, 68–73. 10.16468/j.cnki.issn1004-0366.2022.01.011

[B46] Wang Q. Bao H. F. Ding R. R. Shi Y. W. Yang H. M. Chu M. et al. (2022). Whole-genome sequencing of biocontrol bacterium *Bacillus subtilis* DNKAS and analysis of genes related to biofilm formation. *Herbiv. Livest.* 5 56–64. 10.16863/j.cnki.1003-6377.2022.05.010

[B47] Wang S. Ruan C. Yi L. Deng L. Yao S. Zeng K. (2020). Biocontrol ability and action mechanism of *Metschnikowia citriensis* against *Geotrichum citri-aurantii* causing sour rot of postharvest citrus fruit. *Food Microbiol.* 87:103375. 10.1016/j.fm.2019.103375 31948616

[B48] Wang S. Yang M. Han Y. Sun T. Q. Tang Y. M. Wang L. H. et al. (2025). Isolation and identification of pathogens causing *A. membranaceus* var. mongholicus root rot. *J. Shanxi Agric. Univ.* 45 100–108. 10.13842/j.cnki.issn1671-8151.202503027

[B49] Wang Y. H. Zhao Q. Q. Sun Z. Q. Li Y. H. He H. T. Zhang Y. Y. et al. (2022). Whole-genome analysis revealed the growth-promoting mechanism of endophytic bacterial strain Q2H1 in potato plants. *Front. Microbiol.* 13:1035901. 10.3389/fmicb.2022.1035901 36532474 PMC9751815

[B50] Wang Y. L. Shang M. M. Zhang X. W. Guo X. J. Wang Y. G. (2019). Preliminary study on the inhibitory effect and mechanism of *Pseudomonas* sp. YL11 against *Penicillium expansum*. *Microbiol. China* 46 1081–1091. 10.13344/j.microbiol.china.180470

[B51] Wang Y. Zhao R. Gao Y. Q. Li J. B. Wang C. W. (2026). Control efficiency of complex microbial community on *Astragalus membranaceus* root rot and its impact on rhizosphere microbial community. *Chin. J. Biol. Control.* 42 10–22. 10.16409/j.cnki.2095-039x.2025.02.068

[B52] Wang Z. S. Li Y. Zhao Y. Zhuang L. B. Yu Y. Wang M. Y. et al. (2021). A microbial consortium-based product promotes potato yield by recruiting rhizosphere bacteria involved in nitrogen and carbon metabolisms. *Microb. Biotechnol.* 14 1961–1975. 10.1111/1751-7915.13876 34231972 PMC8449676

[B53] Wang Z. W. Liu X. L. (2008). Medium optimization for antifungal active substances production from a newly isolated *Paenibacillus* sp. using response surface methodology. *Bioresour. Technol.* 99 8245–8251. 10.1016/j.biortech.2008.03.039 18448333

[B54] Wei Q. F. Shi L. W. Wang X. Z. (2020). Effects of four microbial fertilizers applied as basal dressing on the yield of *A. membranaceus*. *Gansu Agric. Sci. Technol.* 6 69–71. 10.3969/j.issn.1001-1463.2020.06.018

[B55] Wu J. Zhu Z. Y. Fu Y. G. Xv Q. F. Yuan Z. L. Lin H. P. (2026). Screening and physiological characteristics of fungi with high siderophore yield and efficient phosphate solubilization. *Mycosystema* 10.13346/j.mycosystema.250350 [Epub ahead of print].

[B56] Xia M. X. Jin J. R. Xiao M. (2020). Study on the relationship between bacterial biofilm and various microbial taxa. *J. Shanghai Norm. Univ.* 49 589–595. 10.3969/J.ISSN.1000-5137.2020.05.012

[B57] Xiong Q. Liu D. Zhang H. H. Dong X. Y. Zhang G. S. Liu Y. P. et al. (2020). Quorum sensing signal autoinducer-2 promotes root colonization of *Bacillus velezensis* SQR9 by affecting biofilm formation and motility. *Appl. Microbiol. Biotechnol.* 104 7177–7185. 10.1007/s00253-020-10713-w 32621125

[B58] Xu Q. (2025). *Identification and Biocontrol of Pathogens Causing Tetradium ruticarpum Fusarium Wilt.* Master’s thesis, China: Chongqing Three Gorges University, 10.27883/d.cnki.gcqsx.2025.000051

[B59] Yan W. B. (2020). *Research on the Growth-Promoting Effect of Paenibacillus terrae on Maize and its Biocontrol Mechanism Against Northern Corn Leaf Blight.* Ph.D. thesis, China: Heilongjiang Bayi Agricultural University, 10.27122/d.cnki.ghlnu.2020.000007

[B60] Yang B. Li Y. X. Xu J. Y. Sha Y. X. (2025). Screening, identification and application of actinomycetes for biocontrol of *Astragalus* root rot. *Chin. J. Biol. Control.* 41 554–560. 10.16409/j.cnki.2095-039x.2025.02.024

[B61] Yang K. Y. Han L. J. Liang Y. P. Huang C. Jiang L. Zhao X. et al. (2023). Siderophore-producing endophytic bacteria in the root nodules of *Lespedeza daurica*: Identification and examination of growth-promoting effect and stress resistance. *Microbiol. China* 50 4413–4432. 10.13344/j.microbiol.china.230089

[B62] Yang S. Yang T. Lin B. Liu X. Z. Xiang M. C. (2018). Screening, identification and evaluation of phosphate-solubilizing efficiency of two phosphate-solubilizing fungi. *Acta Microbiol. Sinica* 58 264–273. 10.13343/j.cnki.wsxb.20170112

[B63] Yang Y. H. (2022). *Study on the Control Effect of Paenibacillus polymyxa KN-03 Bacterium on Citrus Huanglongbing and Optimization of Citrus Tissue DNA Extraction Technology.* Master’s thesis, China: Huazhong Agricultural University, 10.27158/d.cnki.ghznu.2022.002237

[B64] Zhang B. (2024). *Effects of Root Rot on the Quality of Astragalus membranaceus and Preliminary Study on Control Bacteria.* Master’s thesis, China: Lanzhou University of Technology, 10.27206/d.cnki.ggsgu.2024.001097

[B65] Zhang B. Xiao O. L. Wang T. L. Wang D. Zhao H. R. Chen J. Y. et al. (2025). Occurrence regularity and comprehensive control strategies of common diseases in *Astragalus*. *CAB* 41 119–125. 10.11924/j.issn.1000-6850.casb2024-0246

[B66] Zhang J. Guo Q. Yang H. Li X. Pei P. Y. Li Z. G. et al. (2024). Isolation and identification of *Arthrobacter sp*. GCG3 from glycyrrhiza rhizosphere and its antifungal activity and growth-promoting property. *Jiangsu Agric. Sci.* 52 247–255. 10.15889/j.issn.1002-1302.2024.23.033

[B67] Zhang X. C. Zhang H. J. Li S. B. Dong Z. D. Long S. P. Huang X. G. et al. (2024). The control effect of *Trichoderma harzianum* EMF910 on root rot pathogens of *Astragalus membranaceus* in Ningxia saline-alkali regions. *Microbiol. China* 51 4162–4180. 10.13344/j.microbiol.china.231041

[B68] Zhang Y. X. Xu Q. Wang G. J. Shi K. X. (2023). Mixed *Enterobacter* and *Klebsiella* bacteria enhance soybean biological nitrogen fixation ability when combined with rhizobia inoculation. *Soil Biol. Biochem.* 184:109100. 10.1016/j.soilbio.2023.109100

[B69] Zhao X. Hao L. (2020). Effects of *Bacillus amyloliquefaciens* HRH317 on mycelial morphology and ultrastructure of *Fusarium moniliforme*. *Acta Phytophyl. Sin.* 47 110–118. 10.13802/j.cnki.zwbhxb.2020.2019025

[B70] Zheng X. Liu J. Wang X. (2025). Quorum signaling molecules: Interactions between plants and associated pathogens. *Int. J. Mol. Sci.* 26:5235. 10.3390/ijms26115235 40508052 PMC12154563

