# Supplementary material for: Biocontrol mechanisms of two Paenibacillus strains against Astragalus membranaceus root rot and their effects on soil microecological structure
Source: Front Microbiol. 2026 Jun 1;17:1827299. doi: 10.3389/fmicb.2026.1827299 (PMC13265482; doi:10.3389/fmicb.2026.1827299)
Supplement: Supplementary file 1 [file Table_1.docx]

**Supplementary Table S1.** IAA content under different treatments.

| **Treatment** | | **OD_530_** | **IAA production (mg/L)** |
| --- | --- | --- | --- |
| CK | | 0.000±0.000 | 0^c^ |
| HQ-1 | Without adding tryptophan | 0.222±0.005 | 18.316^b^ |
|  | Add tryptophan | 0.412±0.022 | 51.649^a^ |
| HQT-2 | Without adding tryptophan | 0.216±0.029 | 17.261^b^ |
|  | Add tryptophan | 0.386±0.039 | 47.088^a^ |

Values are presented as mean ± SD (n = 3). Different lowercase letters within the same column indicate significant differences among treatments according to Tukey’s HSD test (*p* < 0.05).
