# Supplementary material for: Biocontrol mechanisms of two Paenibacillus strains against Astragalus membranaceus root rot and their effects on soil microecological structure
Source: Front Microbiol. 2026 Jun 1;17:1827299. doi: 10.3389/fmicb.2026.1827299 (PMC13265482; doi:10.3389/fmicb.2026.1827299)
Supplement: Supplementary file 2 [file Table_2.docx]

**Supplementary Table S2.** Main culture media.

| **Culture medium** | **Media component** |
| --- | --- |
| Potato Dextrose Agar (PDA) | 200 g peeled potato, 20 g glucose, 20 g agar, and 1 L water |
| Potato Dextrose Broth (PDB) | 200 g peeled potato, 20 g glucose, and 1 L water |
| King's B medium | 20 g peptone, 10 mL glycerol, 1.5 g K_2_HPO_4_, 1.5 g MgSO_4_•7H_2_O, and 1 L water |
| Phosphate-dissolving detection medium | 10 g glucose, 0.5 g (NH4)_2_SO_4_, 0.3 g NaCl, 0.3 g MgSO_4_, 0.03 g MnSO_4_, 0.03 g K_2_SO_4_, 0.03 g FeSO_4_, 2.5 g Ca_3_(PO4)_2_, 0.2 g lecithin, 18 g agar, and 1 L water |
| Phosphate-solubilizing detection medium | 10 g glucose, 0.5 g (NH4)_2_SO_4_, 0.3 g NaCl, 0.3 g MgSO_4_•7H_2_O, 0.03 g MnSO_4_•H_2_O, 0.3 g KCl, 0.03 g FeSO_4_·7H_2_O, 5 g Ca_3_(PO_4_)_2_, 0.5 g yeast extract, 18 g agar, and 1 L water |
| Potassium-dissolving detection medium | 5 g sucrose, 0.5 g MgSO_4_, 2 g Na_2_HPO_4_, 0.005 g FeCl_3_, 0.1 g CaCO_3_, 0.1 g Bromothymol Blue, 18 g agar, and 1 L water |
| Ashby's Nitrogen-free medium | 0.2 g KH_2_PO_4_, 0.2 g MgSO_4_·7H_2_O, 0.2 g NaCl, 5 g CaCO_3_, 10 g mannitol, 0.1 g CaSO_4_·2H_2_O, 18 g agar, and 1 L water |
| Chrome Azurol S (CAS) medium | 0.0605 g Chrome Azurol S, 0.0729 g Cetyltrimethylammonium Bromide, 0.002645 g FeCl_3_·6H_2_O, 0.29525 g NaH_2_PO_4_·2H_2_O, 1.2135 g Na_2_HPO_4_·12H_2_O, 0.125 g NH_4_Cl, 0.0375 g KH_2_PO_4_, 0.0625 g NaCl, 9 g agar, and 1 L water |
