# Supplementary figures and images for: Biocontrol mechanisms of two Paenibacillus strains against Astragalus membranaceus root rot and their effects on soil microecological structure
Source: Front Microbiol. 2026 Jun 1;17:1827299. doi: 10.3389/fmicb.2026.1827299 (PMC13265482; doi:10.3389/fmicb.2026.1827299)

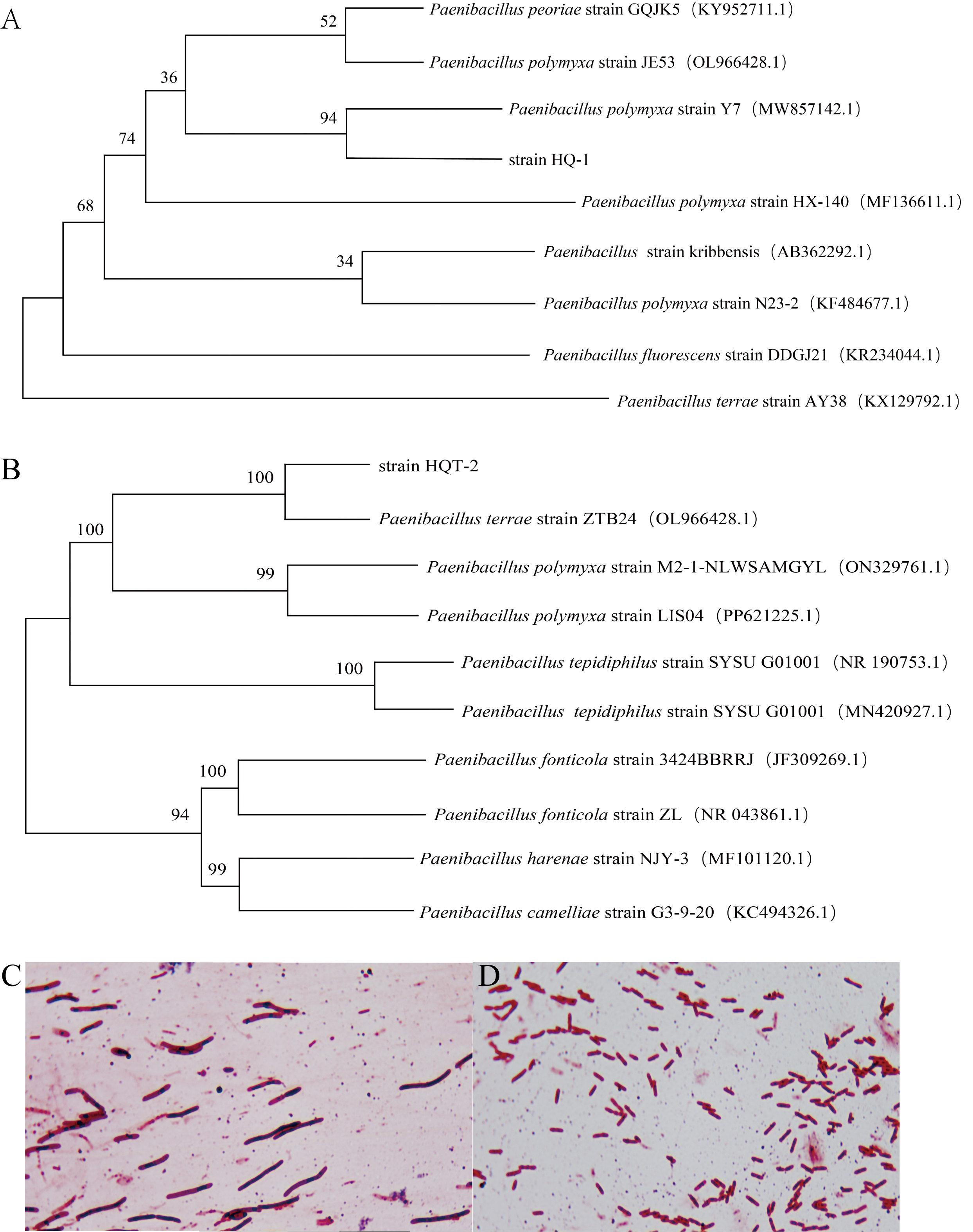

Supplement: Supplementary Figure 1 — Phylogenetic tree of the two biocontrol bacterial strains. (A) HQ-1. (B) HQT-2. (C) Gram staining of HQ-1. (D) Gram staining of HQT-2. [file Image_1.tif]

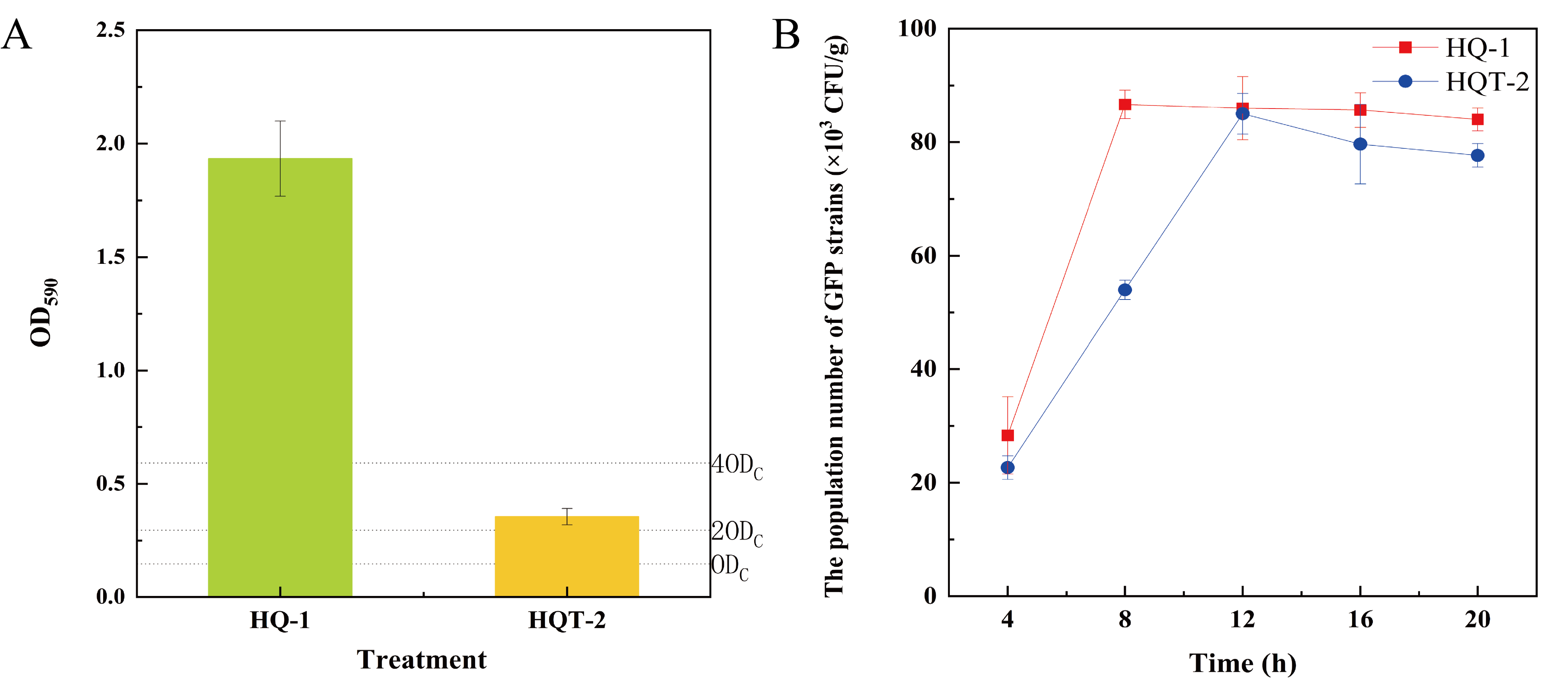

Supplement: Supplementary Figure 2 — (A) Biofilm-forming ability. (B) Hyphal conductivity. [file Image_2.tif]

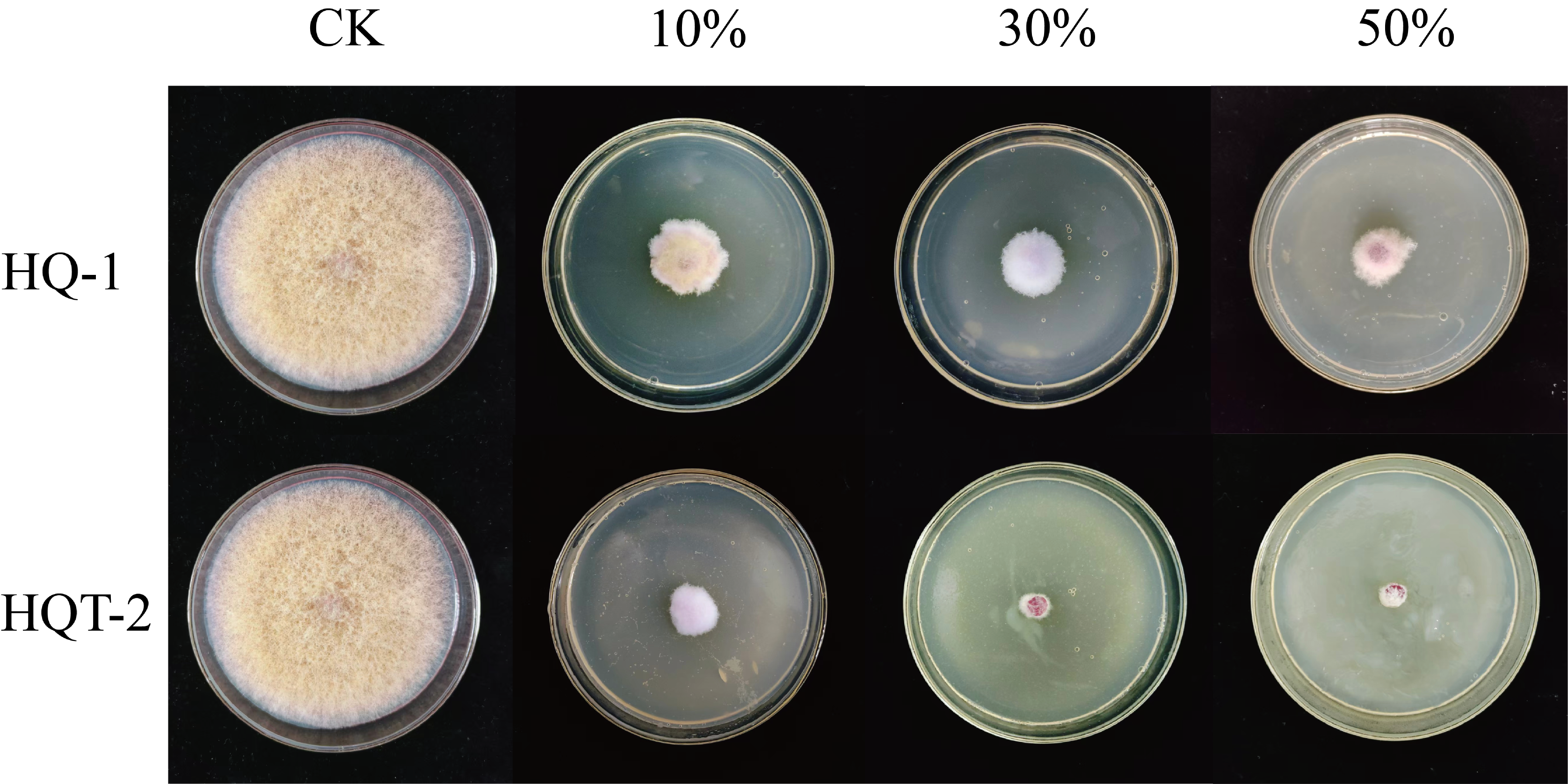

Supplement: Supplementary Figure 3 — Inhibitory effect of cell-free filtrates from the two biocontrol agents on the pathogen. (A) HQ-1. (B) HQT-2. [file Image_3.tif]

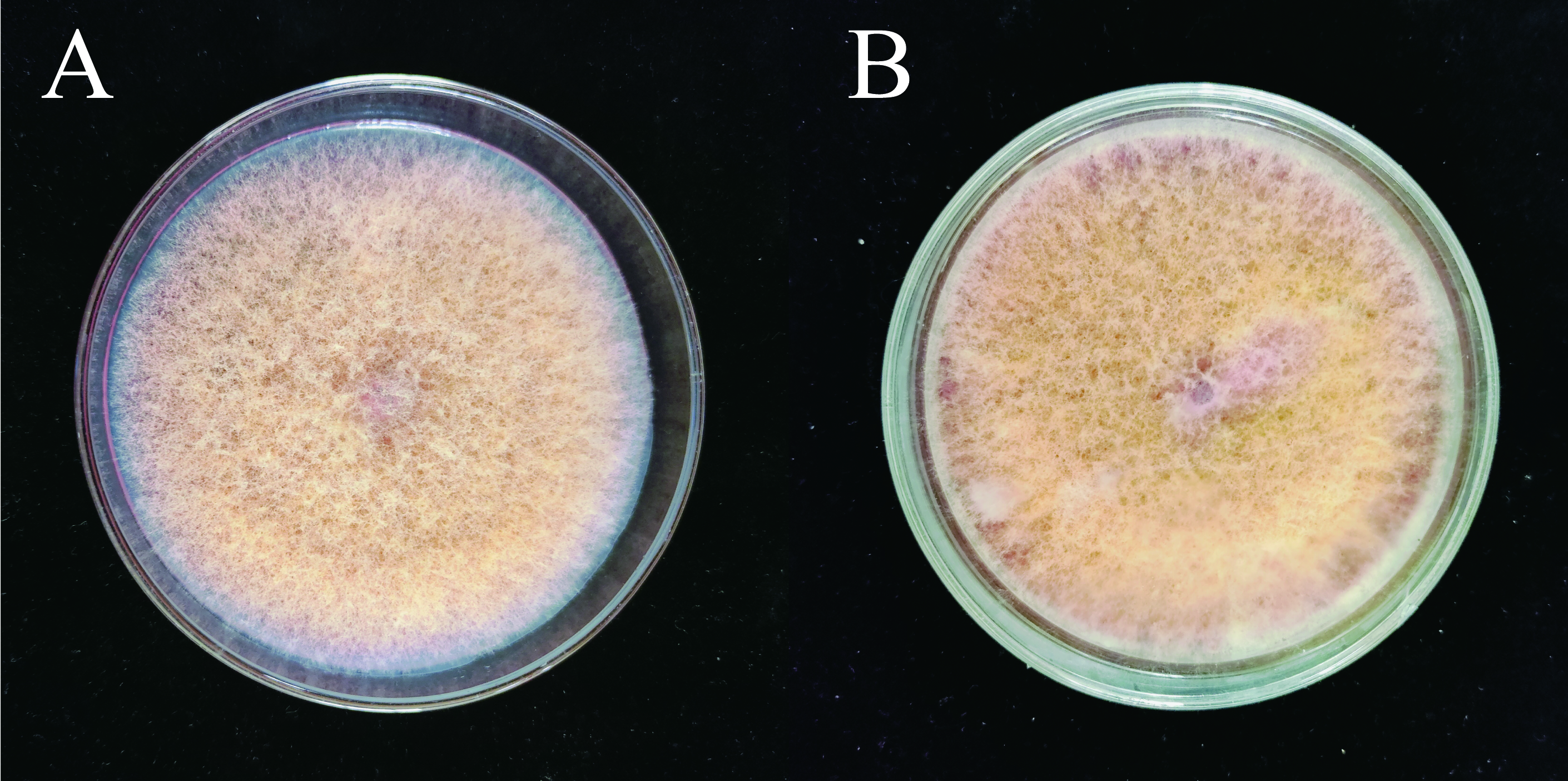

Supplement: Supplementary Figure 4 — Mycelial resuscitation assay. (A) Original strain GF-3. (B) Resuscitated strain GF-3. [file Image_4.tif]

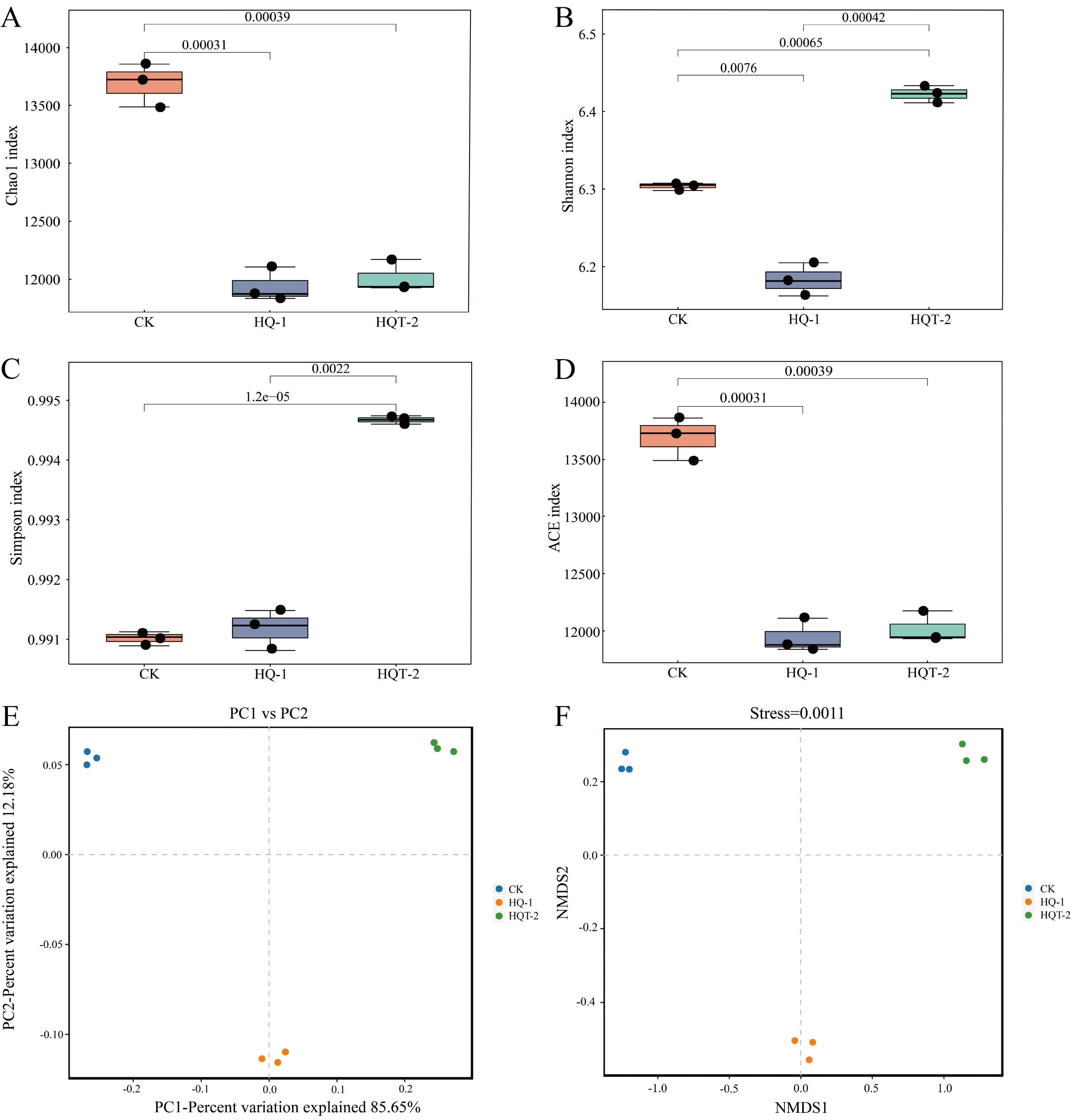

Supplement: Supplementary Figure 5 — Alpha diversity and Beta diversity of the soil microbial community. (A) Chao 1. (B) Shannon index. (C) Simpson index. (D) ACE index. (E) PCA analysis. (F) NMSD analysis. [file Image_5.tif]

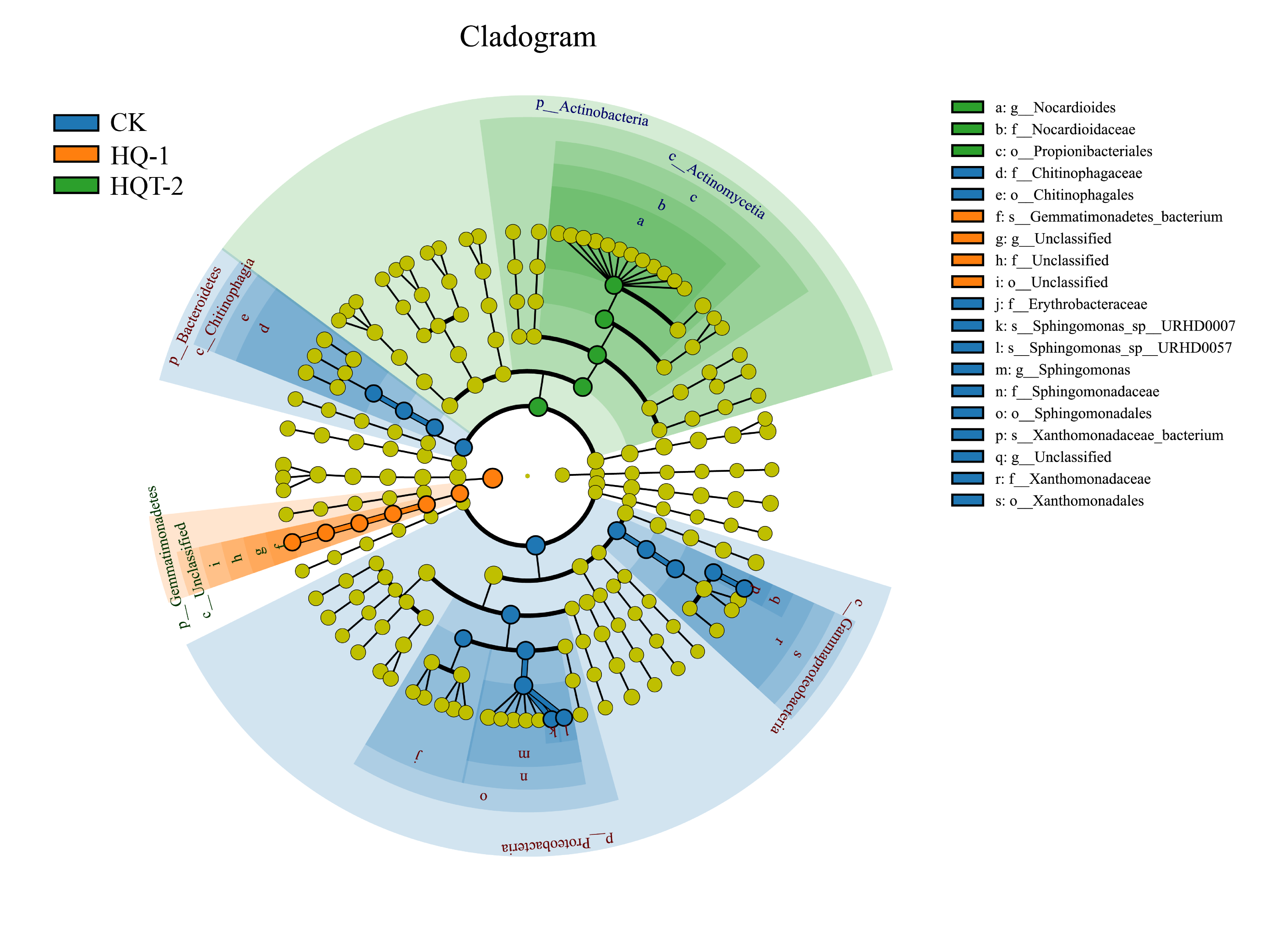

Supplement: Supplementary Figure 6 — Cladogram of intergroup LEfSe analysis. [file Image_6.tif]
